# Oncogenic signaling in the *Drosophila* prostate-like accessory gland activates a pro-tumorigenic program in the absence of proliferation

**DOI:** 10.1242/dmm.052001

**Published:** 2025-04-30

**Authors:** S. Jaimian Church, Ajai J. Pulianmackal, Joseph A. Dixon, Luke V. Loftus, Sarah R. Amend, Kenneth Pienta, Frank C. Cackowski, Laura A. Buttitta

**Affiliations:** ^1^ Molecular, Cellular and Developmental Biology, University of Michigan, Ann Arbor, MI 48109, USA; ^2^ Cancer Ecology Center, The Brady Urological Institute, Johns Hopkins University School of Medicine, Baltimore, MD 21287, USA; ^3^ Karmanos Cancer Institute and Wayne State University, Department of Oncology, Detroit, MI 48201, USA

**Keywords:** Polyploidy, Prostate, Tumorigenesis, *Drosophila* accessory gland, Oncogenic signaling, Postmitotic, Endoreduplication

## Abstract

*Drosophila* models for tumorigenesis have revealed conserved mechanisms of signaling involved in mammalian cancer. Many of these models use highly mitotically active *Drosophila* tissues. Few *Drosophila* tumorigenesis models use adult tissues, when most cells are terminally differentiated and postmitotic. The *Drosophila* accessory glands are prostate-like tissues, and a model for prostate tumorigenesis using this tissue has been explored. In this prior model, oncogenic signaling was induced during the proliferative stages of accessory gland development, raising the question of how oncogenic activity impacts the terminally differentiated, postmitotic adult tissue. Here, we show that oncogenic signaling in the adult *Drosophila* accessory gland leads to activation of a conserved pro-tumorigenic program, similar to that of mitotic tissues, but in the absence of proliferation. In our experiments, oncogenic signaling in the adult gland led to tissue hypertrophy with nuclear anaplasia, in part through endoreduplication. Oncogene-induced gene expression changes in the adult *Drosophila* prostate-like model overlapped with those in polyploid prostate cancer cells after chemotherapy, which potentially mediate tumor recurrence. Thus, the adult accessory glands provide a useful model for aspects of prostate cancer progression that lack cellular proliferation.

## Teaser

**Summary:** The *Drosophila* prostate-like accessory gland is a postmitotic and polyploid tissue. Here, we show similarities between the oncogenic activity in the *Drosophila* accessory gland and in polyploid prostate cancer cells.

## INTRODUCTION

*Drosophila melanogaster* is a convenient and inexpensive animal to model tumorigenesis and cancer (Gong et al., 2021; Ugur et al., 2016). The flies’ short lifespan, low cost of maintenance and conservation of many tumorigenic signaling pathways have led to a robust field of study using *Drosophila* models to investigate the cellular biology and genetics of tumorigenesis across tissues and developmental stages (Dow and Romero, 2010; Moraes and Montagne, 2021; Verheyen, 2022). This is facilitated by tools that allow spatiotemporal control over genetic manipulations, including inducible overexpression, gene knockdown through RNA interference (RNAi), and mosaic loss-of-function or gain-of-function tools (Bangi et al., 2016; Harnish et al., 2021; Sonoshita and Cagan, 2017; Zirin et al., 2020).

Prostate cancer is the second most diagnosed cancer for men worldwide and the third-leading cause of cancer-related death for men over 65 (Culp et al., 2020). Half of men between 70 and 80 years of age show histological evidence of malignancy in the prostate (Carter et al., 1990). The *Drosophila* accessory glands (AGs) serve reproductive functions analogous to those of the mammalian prostate, raising the possibility that these tissues in aged adult animals could be useful models for prostate cancer (Corrigan et al., 2014; Ito et al., 2014; Leiblich et al., 2019; Rambur et al., 2020, 2021; Wilson et al., 2017). The fly reproductive system contains two AGs that release seminal fluid components into the ejaculatory duct, where the seminal vesicles and testes also deposit sperm and additional seminal fluid components (Adams and Wolfner, 2007; Bloch Qazi and Wolfner, 2003; Cridland et al., 2022; Heifetz et al., 2005; Lung et al., 2001; Ravi Ram and Wolfner, 2005; Schnakenberg et al., 2012; Wilson et al., 2017). Fly AGs have a similar, although simpler, epithelial organization to that of the mammalian prostate. The AGs are composed of a polarized secretory epithelial monolayer that forms an apical lumen, surrounded on the basal side by extracellular matrix and a contractile muscle sheath (De Marzo et al., 2010; Rambur et al., 2021; Wilson et al., 2017). The secretory epithelial cells consist of two cell types termed main cells and secondary cells. Main cells comprise the bulk of the secretory epithelium, making up ∼1000 cells per gland lobe, and ∼40-50 large highly secretory secondary cells are located at the distal tip of each gland (Bairati, 1968; Bertram et al., 1992; Sitnik et al., 2016; Susic-Jung et al., 2012; Takashima et al., 2023). The main and secondary cells in the mature adult AG are believed to be largely quiescent. Both of these cell types are known to be polyploid and bi-nucleate owing to variant, truncated cell cycles lacking mitosis that occur during pupal development (Bairati, 1968; Taniguchi et al., 2014, 2018). Recent studies have revealed that there are additional adult-specific endoreplication cell cycles lacking mitoses very early post eclosion and in secondary cells after mating (Box et al., 2024; Leiblich et al., 2019; Sekar et al., 2023). However, unlike the mammalian prostate, this tissue is thought to be completely postmitotic in adults, with no known proliferating or stem cell population.

Prior studies in the *Drosophila* AG have established that these tissues can be used to model important aspects of tumorigenesis (Rambur et al., 2021). Cell growth and migration in the gland are impacted by genes that also play roles in human prostate cancer progression (Ito et al., 2014; Leiblich et al., 2019; Sekar et al., 2023). In addition, activation of Ras/MAPK and Insulin/PI3K signaling, known pathways involved in prostate cancer, could lead to features of neoplastic basal tissue extrusions resembling early stages of metastases in this tissue (Rambur et al., 2020). However, in Rambur et al. (2020), the genetic manipulations leading to pathway activation were performed during the pupal stages of development, specifically prior to the final mitoses and terminal differentiation in the developing AG. Thus, it remained unclear which effects could be due to delays in cell cycle exit or defects in terminal differentiation prior to adulthood. This led us to wonder how the adult tissue would respond to oncogenic-like cell cycle activation after terminal differentiation and cell cycle exit.

Here, we investigated the effects of inducing cell cycle reactivation in the postmitotic adult AG epithelium. Our goal was to investigate the level of cell cycle plasticity and capacity for cell cycle re-entry in AG cells after they had entered quiescence and fully terminally differentiated. We found that adult AG cells can re-enter a non-mitotic endoreduplication cell cycle, which leads to increased cellular hypertrophy and ploidy in the absence of mitosis. Oncogenic signaling or cell cycle re-entry across many cells in the gland led to tissue morphological disruptions and aneuploidy, accompanied by activation of growth and stress signaling pathways, all in the absence of mitoses. Interestingly, transcriptomic analyses revealed a gene expression program that overlaps with other models of tumorigenesis in *Drosophila* in mitotically proliferating tissues, such as the larval imaginal tissues. This indicates that significant aspects of the aberrant signaling observed in these tumor models can occur in the absence of mitoses. We extended our analysis to investigate conserved pathways in non-dividing polyploid cells from mammalian prostate cancer models post chemotherapy. Here, we observed that endocycling prostate cancer cells with increased genomic content (polyaneuploid) exhibit changes in expression of orthologous genes to those we observed in our *Drosophila* model of oncogenic activity in the prostate-like tissue. This suggests that *Drosophila* models of non-proliferating polyploid cells in the adult prostate-like tissue might contribute substantially to understanding prostate cancer cell states even in the absence of cellular proliferation.

## RESULTS

### Oncogenic signaling in the *Drosophila* AG induces epithelial disruption and can induce endocycle re-entry

**Fig. 1.**
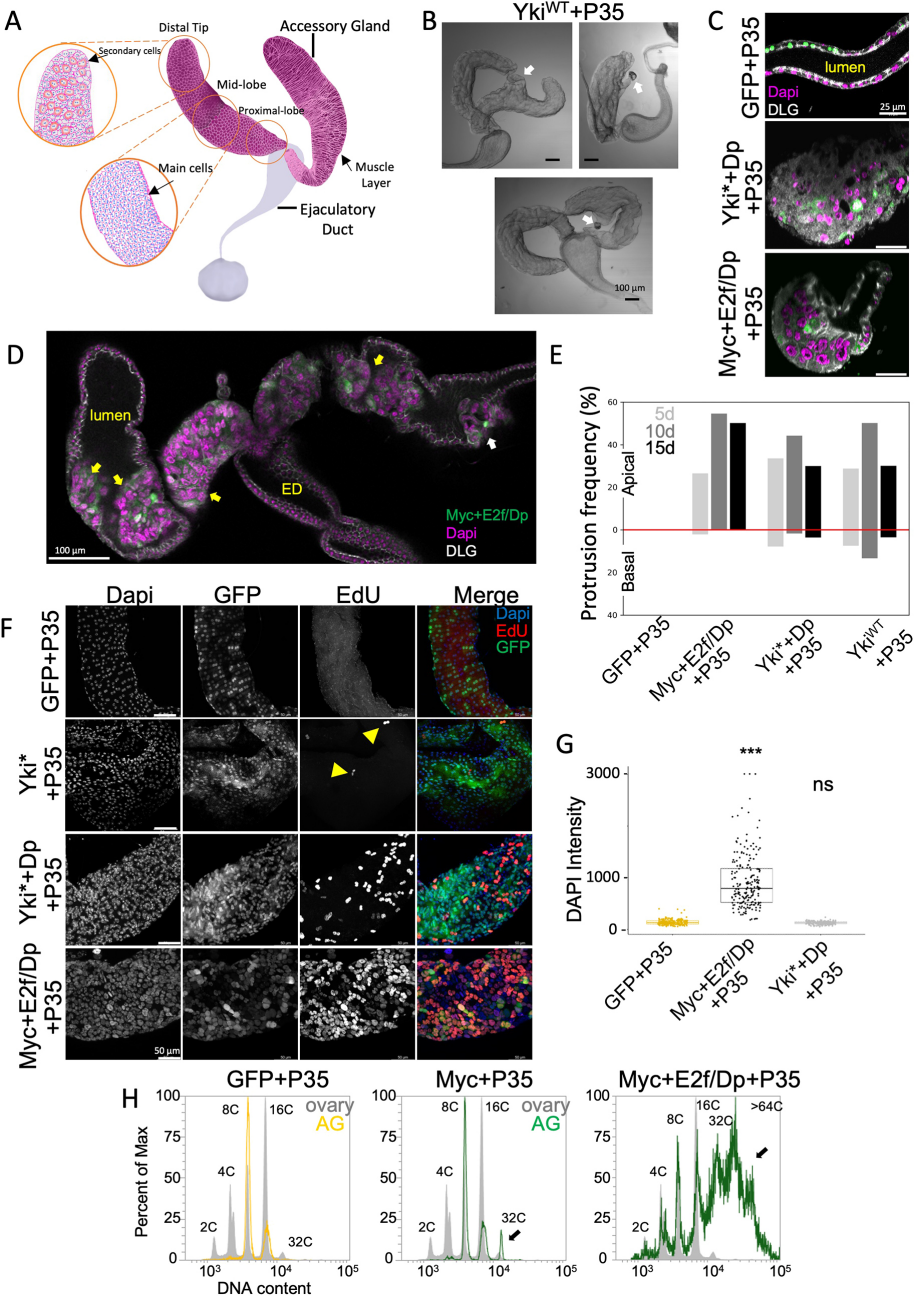
**Oncogenic activation leads to endocycling and tissue hypertrophy in the adult *Drosophila* accessory gland.** (A) Diagram showing the major features of the *Drosophila* accessory gland. (B) Differential interference contrast images of accessory glands with ejaculatory ducts exhibiting basal protrusions (white arrows) when Yki^WT^+P35 is expressed in adults. (C) Optical cross-sections of glands expressing the indicated transgenes for 10 days. DLG, Discs large septate junction protein. The apical lumen is indicated in the control expressing GFP+P35. (D) Optical coronal section through glands and ejaculatory duct (ED) expressing Myc+E2f/Dp+P35 exhibiting apical hypertrophy invading the lumen (yellow arrows) and a basal protrusion (white arrow). (E) Split bar plot showing the frequency of accessory glands with apical or basal protrusions at various time points. d, day. Sample size: GFP+P35 controls, *N*=254; Myc+E2f/Dp+P35, *N*=276; Yki*+Dp+P35, *N*=161; and Yki^WT^+P35, *N*=222. (F) EdU labeling of flies from 6 to 10 days with transgene induction 24-48 h post eclosion. Yellow arrowheads indicate EdU^+^ nuclei. Sample sizes: GFP+P35, *N*=14; Myc+E2f/Dp +P35, *N*=12; Yki*+P35, *n*=4; Yki*+Dp+P35, *N*=13. (G) DAPI integrated density measurements (DAPI intensity) after 10 days of transgene expression. Sample size: 200 nuclei from four glands. (H) Flow cytometry measurements of ploidy from isolated nuclei at 10 days. Gray plots show ploidy measurements from >20,000 ovary nuclei indicating ploidies from 2C to 32C. Black arrows indicate ploidies not present in control glands. At least 5000 nuclei are represented in each histogram. Wilcoxon rank-sum test between control and experimental; for Myc+E2f, *P*<2.2×10^−16^. ns, not significant; ***P≤0.0001.

The *Drosophila* AG is composed of a single-layered, polarized secretory epithelium. The apical cell surface faces the gland lumen, whereas the basal surface is in contact with a collagen-containing basement membrane surrounded by a layer of muscle (Fig. 1A) (Susic-Jung et al., 2012; Rambur et al., 2020). The secretory epithelial cells of the AG are divided into two cell types: main and secondary cells (Fig. 1A). There are ∼40 large secondary cells per AG, located at the distal tip (Fig. 1A). Main cells comprise the remaining ∼1000 secretory cells within each AG. Both cell types in the AG produce various proteins and other molecules that aid in fertility (Bertram et al., 1992; Leiblich et al., 2012; Sharma et al., 2017).

Although widespread genetic changes such as translocations and copy number variations are observed in the prostate, primary tumorigenesis is thought to arise clonally (Wang et al., 2018). As the adult *Drosophila* AG epithelium is polyploid, bi-nucleate, postmitotic and largely quiescent, we utilized a widely used heat-shock-induced ‘flipout’ system to induce transgene overexpression via the Gal4/UAS system in the AG. This approach uses a flippase-induced removal of a stop cassette between an actin promoter and Gal4 coding sequence, allowing UAS-driven gene expression to be activated postmitotically by a heat shock performed in a temporally controlled manner (Pignoni and Zipursky, 1997). The system is also sporadic and titratable, such that the number of cells expressing Gal4 increases with the length of the heat shock. We tested several heat-shock parameters with a goal to induce consistent postmitotic expression in the adult AG epithelia starting 24-48 h post eclosion (Fig. S1). We found the number of AG cells expressing Gal4 using this approach to be variable from animal to animal and highly dependent upon the specific genetic background, with levels varying depending upon which UAS transgenes were expressed, even though all transgenes were crossed into the same hs-flippase (*hsflp*) background. To maximize consistency, all the work shown here used 20 min heat-shock condition, 24-48 h post eclosion. We noted that binucleate main cells are often labeled with Gal4 in neighboring pairs of cells even though the induction event occurs post mitotically (Fig. S1). This is because adult main cells retain ring canals to one neighbor, resulting in pairs of cells that share cytoplasm (Box et al., 2024). One limitation of this approach is the use of an actin promoter to drive Gal4 expression, which leads to the UAS-dependent expression of transgenes in other tissues throughout the adult.

By 4-6 h post eclosion, the polyploid and bi-nucleate AGs epithelial cells exit the cell cycle with two octoploid nuclei (2×8C=16C total cellular DNA content). Under normal conditions, we observe a low level of main cells (<1% per day) continuing to endocycle, generating a small population of cells with two 16C nuclei (32C total cellular DNA content), with most adult AGs epithelial cells in a long-term quiescent state by a few hours post eclosion (Box et al., 2024). Additional endocycles can be driven in main cells by expression of positive cell cycle regulators such as Cyclin D/Cdk4 or Cyclin E (Box et al., 2024; Molano-Fernandez et al., 2022), but, in these instances, the Gal4 driver expresses prior to eclosion. To test whether expression of positive cell cycle regulators could induce cell cycle re-entry in the mature, quiescent adult AG, we overexpressed the *Drosophila* activator E2F complex, composed of E2f1 and Dp (hereafter referred to as E2f/Dp), a known driver of the endocycle (Zielke et al., 2011), or *Drosophila* Myc, a pro-growth regulator that promotes cell cycle entry and endocycling (Pierce et al., 2004). To prevent the elimination of aberrantly cycling cells, we also co-expressed the *Drosophila* apoptosis inhibitor, baculoviral P35 (Hay et al., 1994). We observed minimal effects on overall AG size or morphology with E2f/Dp or Myc expression, although we did observe S-phases via 5-ethynyl-2'-deoxyuridine (EdU) incorporation in a fraction of main cells (up to 5% of cells), demonstrating that quiescent main cells can be readily induced to re-enter the cell cycle in adults through ectopic Myc or E2f/Dp activity (Fig. S2A).

To activate oncogenic signaling associated with cell cycle re-entry and overgrowth, we increased the expression of wild-type Yorkie (Yki^WT^), the fly ortholog of YAP1/TAZ, the key transcriptional regulator downstream of the Hippo pathway responsible for controlling organ size (Dong et al., 2007). When Yki^WT^ was overexpressed in the adult AG, we occasionally observed abnormal masses basally protruding from the gland (Fig. 1B). Additionally, cyst-like protrusions were occasionally observed, most often at the distal tips of basal protrusions (Fig. 1B, right and bottom panels). To see whether this phenotype could be recapitulated in other oncogenic signaling contexts, we combined Myc and E2f/Dp expression simultaneously or attempted to combine overexpression of E2f/Dp with a constitutively active form of Yki, referred to as Yki* in our study (Oh and Irvine, 2009). However, the Yki*+E2f/Dp line lost efficient UAS-E2f induction, and hereafter is referred to as Yki*+Dp. When Myc+E2f/Dp or Yki*+Dp were expressed in the adult AG, we rarely observed tissue basally protruding from the gland, but very frequently observed apically protruding or invading cell masses filling or occluding the gland lumen (Fig. 1C,D; Fig. S2D). We quantified the frequency of gland alterations under Myc+E2f/Dp-, Yki*+Dp- and Yki^WT^-expressing conditions for 5-15 days (Fig. 1E). Regardless of genotype, most epithelial disruptions invaded the lumen apically, with 50% or more of animals across genotypes exhibiting apical protrusions or invasions of cells. One limitation of our time course quantification is that we noted significant decreases in animal viability after 10 days in the genotypes overexpressing E2f/Dp or Dp. This suggests that the most-affected animals had died by day 10, possibly owing to cell cycle disruptions across various tissues.

The additional tissue overgrowth apically and basally prompted us to examine glands for evidence of mitotic proliferation via staining for Phosphorylated histone H3 Ser10 (PH3). In total, we examined over ∼150 samples across genotypes and timepoints and found no evidence of any mitotic labeling (Fig. S3). However, we observed extensive S-phases via a 4-day EdU feeding protocol (from day 6-10) in 70-100% of animals co-expressing Myc+E2f/Dp or Yki*+Dp with P35 (Fig. 1F). By comparison, we observed no significant EdU labeling in control animals expressing P35 and GFP only, and rare EdU labeling of a few binucleated cells in glands expressing Yki* without Dp. At the 10-day time point, we also measured the 4',6-diamidino-2-phenylindole (DAPI) nuclear intensity for 200 nuclei per sample from four different AGs per genotype (Fig. 1G). As expected, we found evidence of increased nuclear DNA content in Myc+E2f/Dp conditions, whereby EdU^+^ nuclei were visibly larger, while Yki*+Dp-expressing nuclei appeared more normal (Fig. 1G). This suggests that Myc+E2f/Dp expression led to multiple rounds of endocycles during the assay, whereas Yki*+Dp expression induced widespread endocycle re-entry, but with only limited rounds of additional endocycling. To confirm this, we performed flow cytometry on AG nuclei at 10 days post Gal4 induction (Fig. 1H). Ovaries were used as a ploidy ‘ladder’ to place 2C, 4C, 8C and 16C peaks using the stereotypical double peak at 4C (Bosco et al., 2007; Calvi et al., 1998; Lilly and Spradling, 1996). In control 10-day-old animals expressing only GFP and P35, we observed mostly octoploid nuclei with some 16C nuclei, consistent with recent work (Box et al., 2024). In Myc+P35 conditions, we observed a small 32C population of nuclei appear, consistent with the penetrant but limited EdU labeling observed in this condition (Fig. 1H; Fig. S2C). By contrast, many nuclei from cells expressing Myc+E2f/Dp with P35 had a DNA content well beyond 32C and exhibited intermediate DNA ploidies indicative of aneuploidy. Glands expressing Yki* exhibited mostly 8-16C nuclei, whereas glands expressing Yki*+Dp exhibited a shift to higher ploidy, consistent with limited endoreplication, as observed by EdU labeling (Fig. S2A). This suggests that the basal and apical tissue protrusions induced by Yki^WT^ and Yki*+Dp expression are likely caused largely by epithelial remodeling, while the protrusions induced by Myc+E2f/Dp expression may involve tissue hypertrophy through sustained re-entry into the endocycle.

### Oncogenic signaling in the adult AG affects epithelial organization

**Fig. 2.**
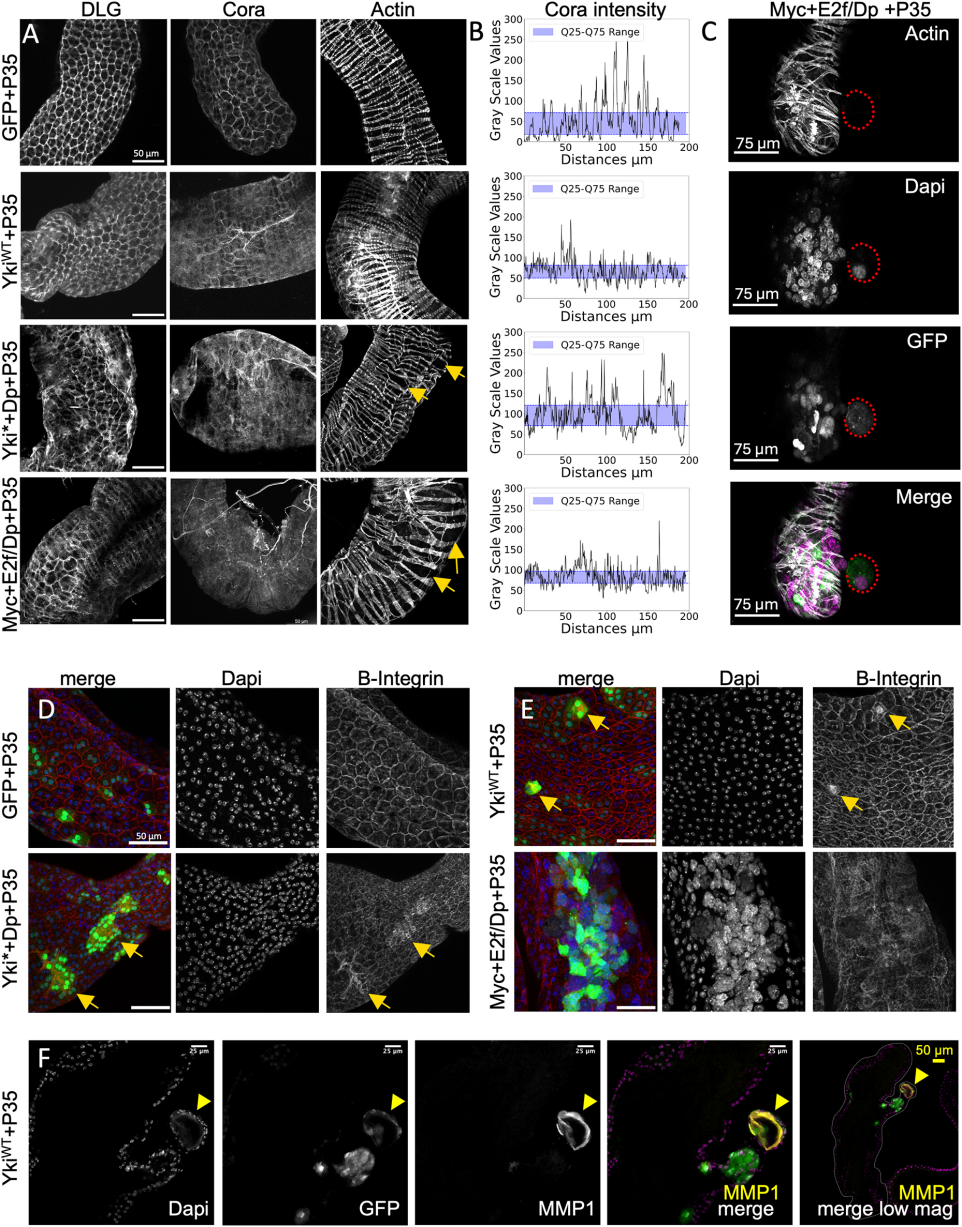
**Oncogenic activation leads to epithelial remodeling in the adult *Drosophila* accessory gland.** (A) Discs large (DLG) and Coracle (Cora) septate junction proteins become mislocalized in adult glands after 15 days of oncogenic transgene expression. Labeling Actin with Phalloidin reveals large gaps in the muscle surrounding the accessory gland under oncogene expression conditions (yellow arrows). Sample sizes for DLG: GFP+P35, *N*=8; Myc+E2f/Dp+P35, *N*=12; Yki*+Dp+P35, *N*=6. Sample sizes for Cora: control, *N*=6; Myc+E2f/Dp, *N*=11; Yki*+Dp, *N*=4. Sample sizes for Actin/Phalloidin: control, *N*=21; Myc+E2f/Dp, *N*=27; Yki*+Dp, *N*=15. (B) Line scans of Cora intensity for the images in A. The shaded blue area shows the interquartile range. Q, quartile. (C) A basal extrusion in a gland expressing Myc+E2f/Dp+P35 for 5 days that extrudes beyond the muscle layer (red dotted outline). (D,E) Beta (B)-integrin (Mys) enrichment (yellow arrows) in glands expressing the indicated transgenes for 10 days. Sample sizes: Yki*+Dp, *N*=12; Yki^WT^, *N*=8; GFP+P35, *N*=8; Myc+E2f/Dp+P35, *N* =11. Scale bars: 50 μm. (F) An example of a basal protrusion in a gland expressing Yki^WT^+P35 with a basal cyst-like extrusion at the tip (yellow arrowheads) positive for Mmp1. Note that this is the fluorescence image corresponding to the gland in Fig. 1B (top-right panel).

We noted that glands exhibiting overgrowth with apical and basal protrusions also exhibited altered localization of the septate junction proteins, Discs large (DLG; also known as Dlg1) (Fig. 1D, Fig. 2A; Fig. S2B), Coracle (Cora) (Fehon et al., 1994; Woods and Bryant, 1993) (Fig. 2A) and Fasciclin 3 (Fas3) (Fig. S4). We observed significant mislocalization of DLG and Cora and confirmed mislocalization of Cora at 15 days using line scans of fluorescence intensity across cell–cell junctions. Cora levels at cell–cell junctions (peaks) were reduced, whereas non-junctional staining was increased (valleys) in Myc+E2f- or Yki*+E2f-expressing animals (Fig. 2B). We next examined whether the actin muscle layer was disrupted. Staining for actin using fluorophore-conjugated phalloidin revealed increased gaps in the muscle fibers in Yki*+Dp or Myc+E2f/Dp conditions (Fig. 2A, yellow arrows). We observed cyst-like basal extrusions poking through one of the gaps in the muscle layer, suggesting that the cyst-like structures extrude basally outside the epithelium (Fig. 2C).

The cyst-like extrusions are reminiscent of a neoplastic-like phenotype in the AG previously reported under conditions of oncogenic Ras^V12^ expression induced during proliferative AG stages and into adulthood (Rambur et al., 2020). With oncogenic Ras overexpression, this phenotype was reported at a frequency of 20-80% of glands. In our conditions, we observed basal extrusions much more rarely, in 2-15% of glands depending on timepoint and genotype (Fig. 1D), with the caveat mentioned previously that our most-affected animals die at later time points owing to actin-Gal4-driven expression in other tissues. This suggests that the developmental timing of the oncogenic induction could have an important impact on the penetrance of the basal extrusion phenotype and that more mature, terminally differentiated AG cells are more refractory to developing this phenotype, even under strong re-activation of the cell cycle. Consistent with this, when we activated Ras^V12^ expression in adult glands 24-48 h post eclosion, we never observed any basal extrusions (0/15 animals). This indicates that oncogenic Ras does not induce a neoplastic-like phenotype in the AG unless the expression is induced when cells are in a state of mitotic proliferation prior to cell cycle exit.

### Basal extrusions exhibit different signaling pathway activation than hypertrophic overgrowths

The presence of cyst-like basal extrusions and their association with basal epithelial protrusions led us to consider that apically or basally protruding cells may exhibit early metastatic phenotypes such as increased integrin expression and expression of matrix metalloproteinases to degrade the extracellular matrix. Indeed, we observed increased Beta-integrin (also known as Mys) staining (Fig. 2D) as well as increased Mmp1 expression in our oncogenic conditions (Fig. S4). Mmp1 expression was particularly pronounced on cyst-like extrusions at the tip of basal protrusions in the Yki^WT^ overexpression condition (Fig. 2F; note that this extrusion example is also shown in Fig. 1B). The development of protrusions extending from the mid-lobe region of the AGs resembled extra-prostatic extensions observed in human prostate cancer associated with YAP1 overexpression (Collak et al., 2017).

**Fig. 3.**
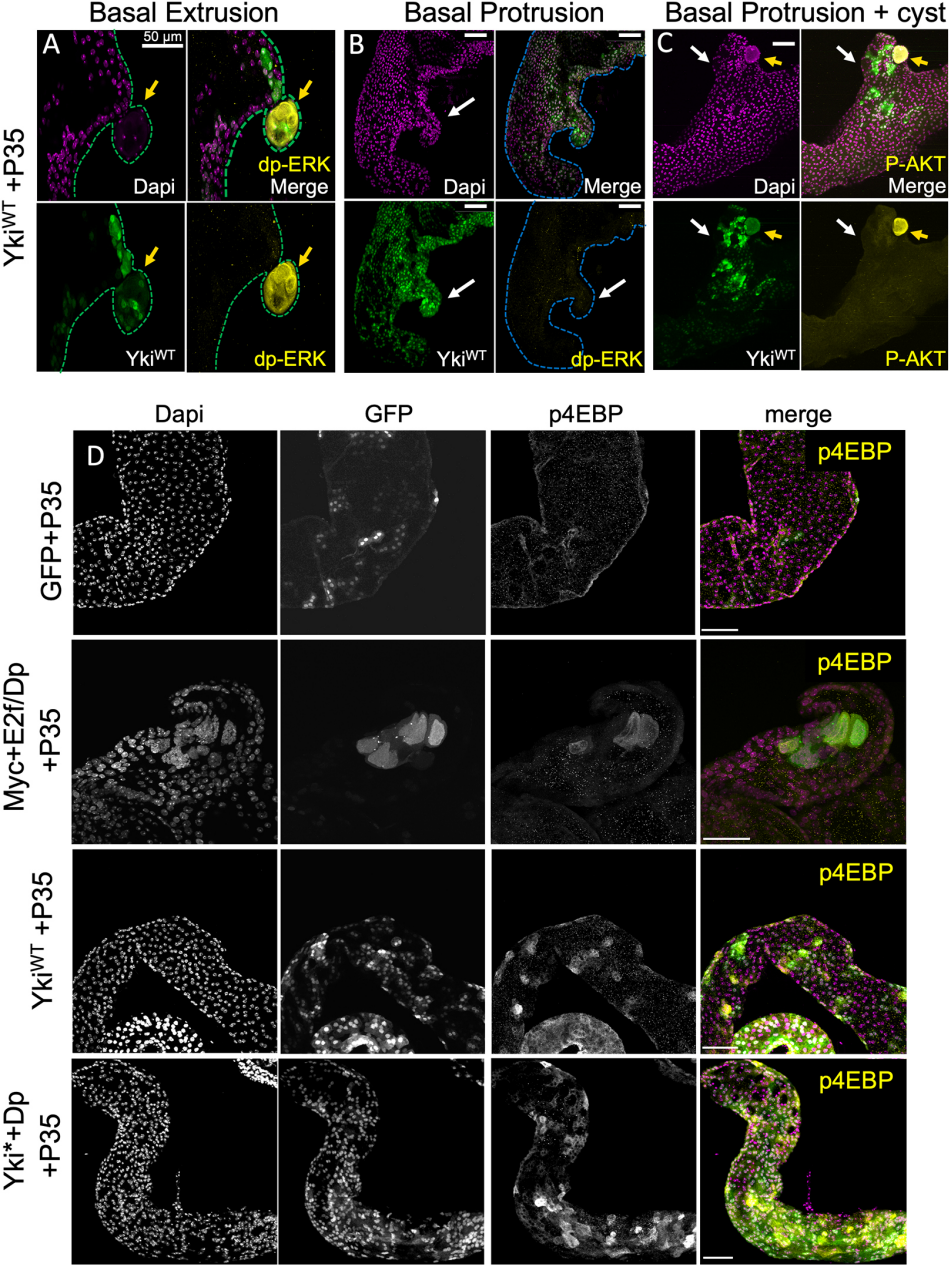
**Signaling pathway activity in hypertrophic protrusions versus basal extrusions.** (A-C) Yki^WT^+P35-expressing glands at 20 days exhibit a range of phenotypes including basal extrusion (A), basal protrusions (B) and basal protrusions with cyst-like extrusions (C). Basal extrusions including cyst-like extrusions (yellow arrows) are positive for dp-ERK (A) and pAkt (C), whereas basal protrusions that remain within the epithelia are not (white arrows). Sample size: *N*=14. (D) Oncogenic activity leads to increased p4EBP after 10 days of transgene expression. Sample sizes: GFP+P35, *N*=12; Myc+E2f/Dp+P35, *N*=8; Yki*+Dp, *N*=10. Scale bars: 50 µm.

We next compared the signaling pathways activated in our oncogenic contexts to those in cells expressing oncogenic hyperactive Ras^V12^ in basal extrusions (Rambur et al., 2020). Ras^V12^ overexpression in the adult AG induced high levels of di-phosphorylated ERK (also known as Rolled) (dp-ERK) and phosphorylated Akt (pAkt) (Fig. S5). Myc+E2f/Dp-, Yki*+Dp- or Yki^WT^-expressing glands were negative for high levels of dp-ERK and pAkt (Fig. S5), except in instances when basal cyst-like extrusions occurred. In these cases, the extrusions were positive for dp-ERK and pAkt, whereas epithelial protrusions remained negative (Fig. 3; Fig. S5). Expression of Myc+E2f/Dp, Yki*+Dp or Yki^WT^ was sufficient to activate growth signaling via phosphorylated 4EBP (also known as Thor) (p4EBP), a target of the TORC1 complex downstream of PI3K signaling, with or without obvious cellular hypertrophy (Fig. 3D; Fig. S5). This suggests that oncogenic gene expression in non-proliferative adult AGs induces epithelial reorganization and hypertrophic growth through TORC1 signaling, but without activating the TORC2/Akt signaling branch (Frappaolo and Giansanti, 2023), unless additional events such as developmental proliferation or basal extrusion leading to MAPK signaling also occur. Most of the cyst-like basal extrusions we observed with adult activation of oncogenic signaling developed on basal epithelial protrusions. This suggests that the epithelial remodeling leading to basal protrusions ultimately leads to basal extrusions that exhibit additional signaling re-activation in adult glands. Our examination of the cell cycle in these conditions suggests that this progression can occur in the absence of proliferation and even under conditions of limited endocycling.

### A shared tumor signaling network is activated in postmitotic tissues, even in the absence of proliferation

**Fig. 4.**
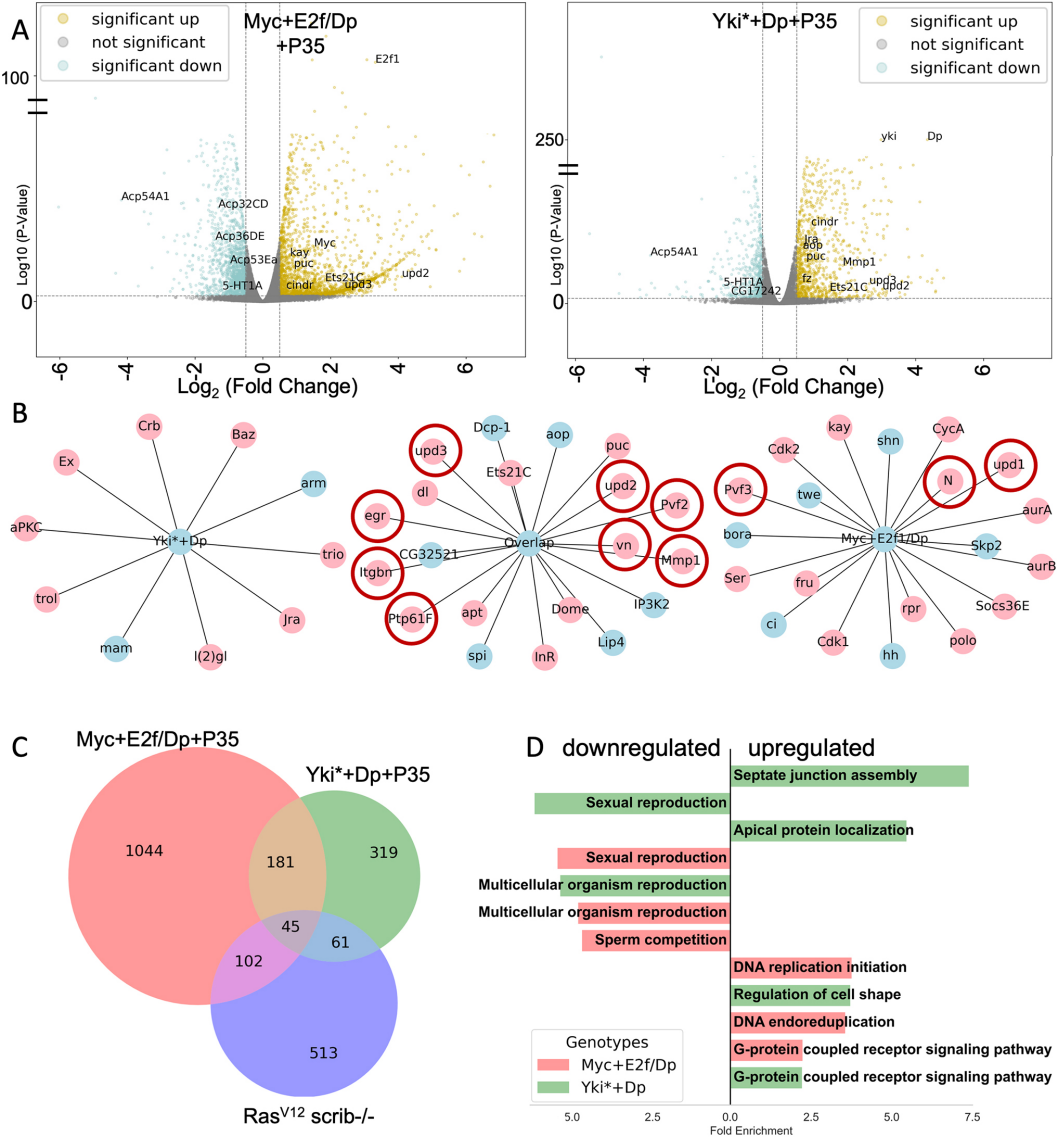
**Activation of a common tumorigenic program in the absence of mitoses.** (A) Volcano plots of RNA-sequencing data for glands expressing Myc+E2f/Dp+P35 and Yki*+Dp+P35 for 10 days compared to GFP+P35 controls. Transcripts significantly upregulated (gold) and downregulated (cyan) are shown. (B) Examples of genes commonly upregulated in *Drosophila* tumor models curated from the literature in Table S5. Genes in pink overlap with the *Ras^V12^,scrib^−/−^* dataset from Floc'hlay et al. (2023). Genes circled in red were selected for testing ortholog upregulation in mammalian cell culture. Genes in blue are upregulated in the accessory glands expressing oncogenes and previously associated with *Drosophila* tumor and wound healing models. Line distances to central nodes are arbitrary and were used to distribute gene names for readability. (C) Overlap of oncogene-induced accessory gland gene expression with a commonly used *Ras^V12^,scrib^−/−^* larval mitotic tumor model (Floc'hlay et al., 2023). (D) Gene Ontology term analysis via DAVID biological process categorization.

The expression of Mmp1 and Beta-integrin, and phosphorylation of TORC1 targets, suggested that our Yki*+Dp-, Yki^WT^- and Myc+ E2f/Dp-expressing tissues are activating a shared tumorigenic program in the absence of mitotic proliferation. To test this, we performed RNA sequencing (RNAseq) on our Yki*+Dp, Yki^WT^ and Myc+E2f/Dp versus control animals expressing GFP+P35 at the 10-day time point to identify shared gene expression changes. We visually confirmed similar levels of transgene expression using the UAS-GFP levels prior to RNA isolation for each sample (i.e. we selected glands with >1/3 of cells GFP^+^). We confirmed induction of the expected transgenes (Fig. S6), and observed a suite of upregulated and downregulated genes in each condition for the cell cycle, responses to stress, and reproduction and fertility (Fig. 4A; Figs S7 and S8).

In both Yki*+Dp and Myc+ E2f/Dp conditions, we observed upregulation of several genes known to be upregulated in wound healing and tumorigenic models in flies, including JNK (also known as Bsk in *Drosophila*) signaling targets *Ets21C*, *Mmp1*, *puckered* and hyperplasia-inducing JAK (also known as Hop in *Drosophila*)/STAT (also known as Stat92E in *Drosophila*) signaling pathway ligands *upd2* and *upd3* (Fig. 4A,B). We also observed the downregulation of AG-specific genes (such as those encoding many Accessory gland proteins) and genes involved in fertility, mating behavior and sperm competition (Fig. 4A,D). This suggests that activation of oncogenic pathways and cell cycle re-entry occur at the expense of AG function (see downregulation of reproduction-associated genes in Fig. 4A and Fig. S7).

We observed a strong overlap between the Yki*+Dp and Myc+ E2f/Dp gene expression signatures that converge on several genes known to be commonly misregulated in proliferative tumor models in various larval tissues, including imaginal discs and brains. We created a node-based graph depicting the common *Drosophila* tumor-misregulated genes in our oncogenic backgrounds and the overlapping gene set between both datasets (Fig. 4B). We also observed stronger misregulation of cell cycle-related E2f targets (e.g. *CycA*, *Cdk2*, *aurA*, *aurB*, *polo*) in the Myc+E2f/Dp signature that is not as strong in the Yki*+Dp gene expression signature (Fig. 4B), although Yki*+Dp did upregulate several cell cycle genes (*Mcm7*, *tefu*, *RnrL*, *RecQ4*, *PolA1*) consistent with the limited endocycling observed. The strong induction of E2f1 in the Myc+E2f/Dp line and the positive feedback regulation between E2f and Myc and their synergistic roles in regulating cell cycle entry from G1 led to a strong DNA replication signature (Fig. S9) (Duman-Scheel et al., 2004; Fernandez et al., 2003; Leone et al., 1997; Matsumura et al., 2003). By contrast, in the Yki*+Dp gene expression signature, we observed stronger misregulation of genes involved in cell polarity, cell–cell adhesion complexes and cytoskeletal regulators (e.g. *arm*, *aPKC*, *baz*, *crb*, *ex*) (Fig. 4B; Fig. S9), consistent with the known roles of the Hippo signaling pathway in regulating and responding to epithelial polarity, cellular adhesion and tissue tension (Boggiano and Fehon, 2012; Borreguero-Munoz et al., 2019; Doggett et al., 2011; Elbediwy et al., 2016; Kroeger et al., 2024; Ling et al., 2010; Richardson and Portela, 2017; Sarpal et al., 2019; Su et al., 2017; Yang et al., 2015). We also observed significant upregulation of *yki* expression in Myc+E2f/Dp-expressing glands, suggesting that Yki could be engaged in epithelial remodeling in both oncogenic conditions.

Oncogenic gene expression in the AG engages common tumorigenic gene networks described in other tumor models, even in the absence of mitosis (Hamaratoglu and Atkins, 2020; Khan et al., 2013; Logeay et al., 2022). In particular, we observed significant overlap with a gene expression signature from the *Drosophila* metastatic tumor model of oncogenic Ras combined with the loss of epithelial polarity through loss of the Scribble (Scrib) protein (*Ras^V12^,scrib^−/−^* mutant) (Fig. 4C) (Atkins et al., 2016; Kulshammer et al., 2015) and wound healing gene expression profiles (Khan et al., 2017) (Table S5). The overlap with wound healing and tumorigenic signatures encompasses genes involved in promoting proliferation (*Jra/kay*, Pvf, *trbl*, Upd) as well as genes more recently described to be part of an aberrant ‘pro-senescence’ program induced by JNK signaling present in wounding and tumorigenic models (*Mmp1*, *Ptp61F*, *Socs36e*) (Floc'hlay et al., 2023). This demonstrates that genes involved in cancer hallmarks can be activated in the adult secretory AG epithelium, even in the absence of proliferation.

### Overexpression of the ERG ortholog Ets21C drives cell cycle re-entry in the adult AG and synergizes with Myc to increase DNA replication

In our RNAseq study, the *ERG* ortholog and JNK target *Ets21C* was significantly upregulated in both Myc+E2f/Dp and Yki*+Dp conditions. Ets21C has previously been shown to transcriptionally activate cell cycle genes, including many E2f-regulated targets (Zhang et al., 2022). We therefore wondered whether Ets21C overexpression could drive endocycle re-entry in the quiescent adult AG and how it compared to E2f or Myc overexpression.

**Fig. 5.**
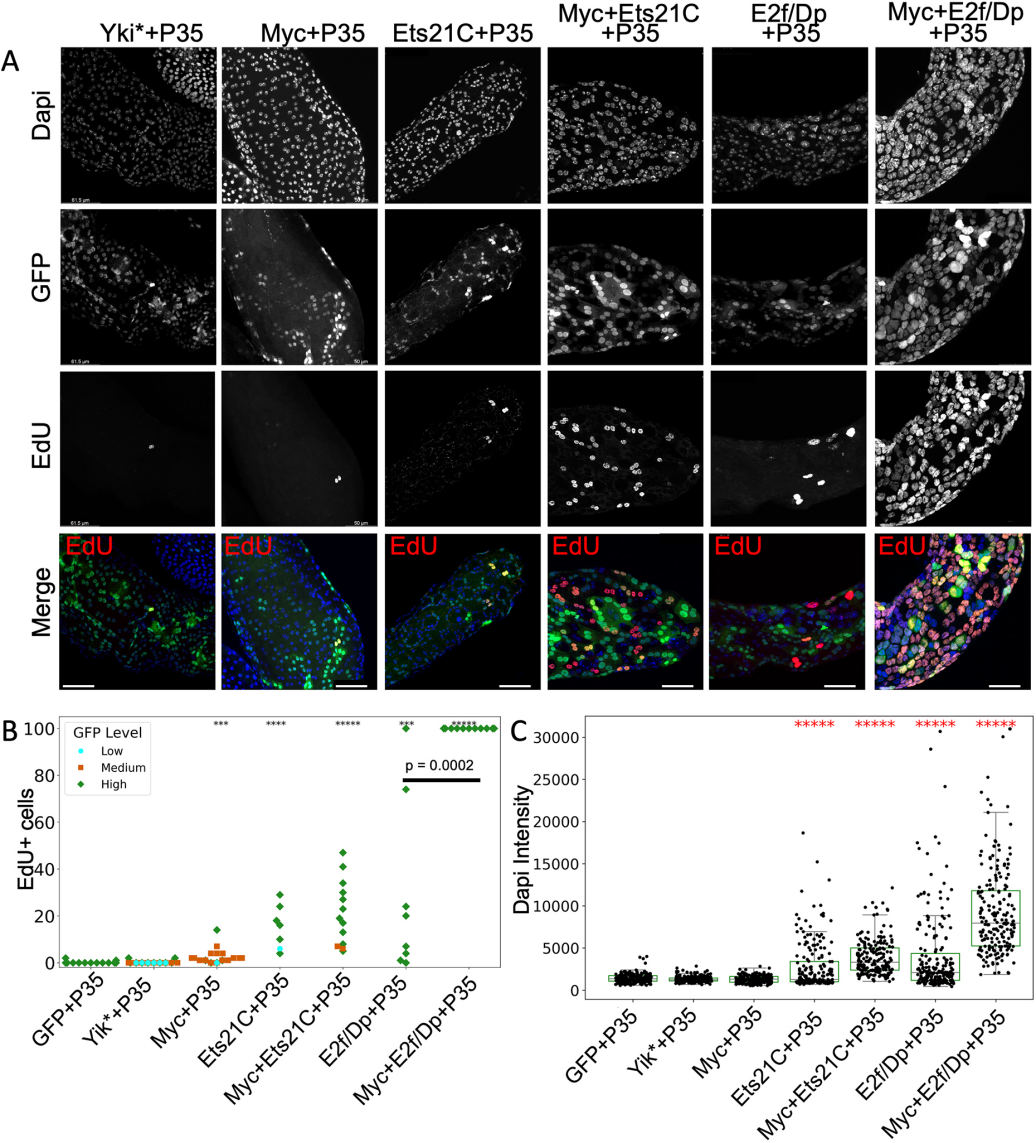
**Ets21C overexpression in the *Drosophila* accessory gland leads to increased DNA replication and aneuploidy.** (A) EdU assays for DNA replication in adult glands from 6 to 10 days of expression of the indicated transgenes. Sample sizes: GFP+P35, *N*=14; Myc+P35, *N*=22; Ets21C+P35, *N* =7; Myc+Ets21C+P35, *N*=5; E2f/Dp+P35, *N*=7; Myc+E2f/Dp+P35, *N*=12; Yki*+P35, *N*=8. Scale bars: 50 µm. (B) Quantification of EdU^+^ cells for the indicated genotypes; the dot color indicates the level of GFP expression based on the scale shown in Fig. S1C-F. Samples with >100 EdU^+^ cells were plotted at a maximum of 100. Note that every sample from the Myc+E2f/Dp+P35 condition has >100 labeled cells. (C) DAPI intensity quantifications from 200 randomly selected nuclei for four biological replicates for 10-day-old samples of the indicated genotypes. For EdU comparisons, we applied the two-sided Wilcoxon–Mann–Whitney test comparing experimental genotypes against the control (GFP+P35) condition. ***P≤0.001, ****P≤0.0001, *****P≤0.00001.

A 4-day EdU labeling assay was conducted to assess endocycle re-entry from days 6-10 across various conditions: Yki*, Myc without E2f, Ets21C, Ets21C+Myc, E2f/Dp and Myc+E2f/Dp (Fig. 5A). Observations indicated no or few EdU-labeled cells under control conditions (*hsflp* with only *UAS-GFP* and *P35*) and for Yki* (Fig. 5B). Myc overexpression resulted in a modest, yet statistically significant, increase in EdU-labeled cells compared to the control, reflecting an increase from approximately one cell per ten animals to an average of one cell per animal (Fig. 5B). By contrast, increased EdU labeling was observed in conditions expressing Ets21C, Myc+Ets21C, E2f/Dp and Myc+E2f/Dp. When combined with Myc, both E2f/Dp and Ets21C expression induced an increased number of EdU^+^ cells per animal as well as increased nuclear DNA content (Fig. 5C). We suggest that, as described in the *Drosophila* intestine, high levels of Ets21C expression act in a manner similar to high E2f activity to induce cell cycle genes involved in endocycling and DNA replication (Zhang et al., 2022), which is enhanced and potentiated by co-expression with Myc.

### High Ets21C promotes non-autonomous increases in nuclear size

**Fig. 6.**
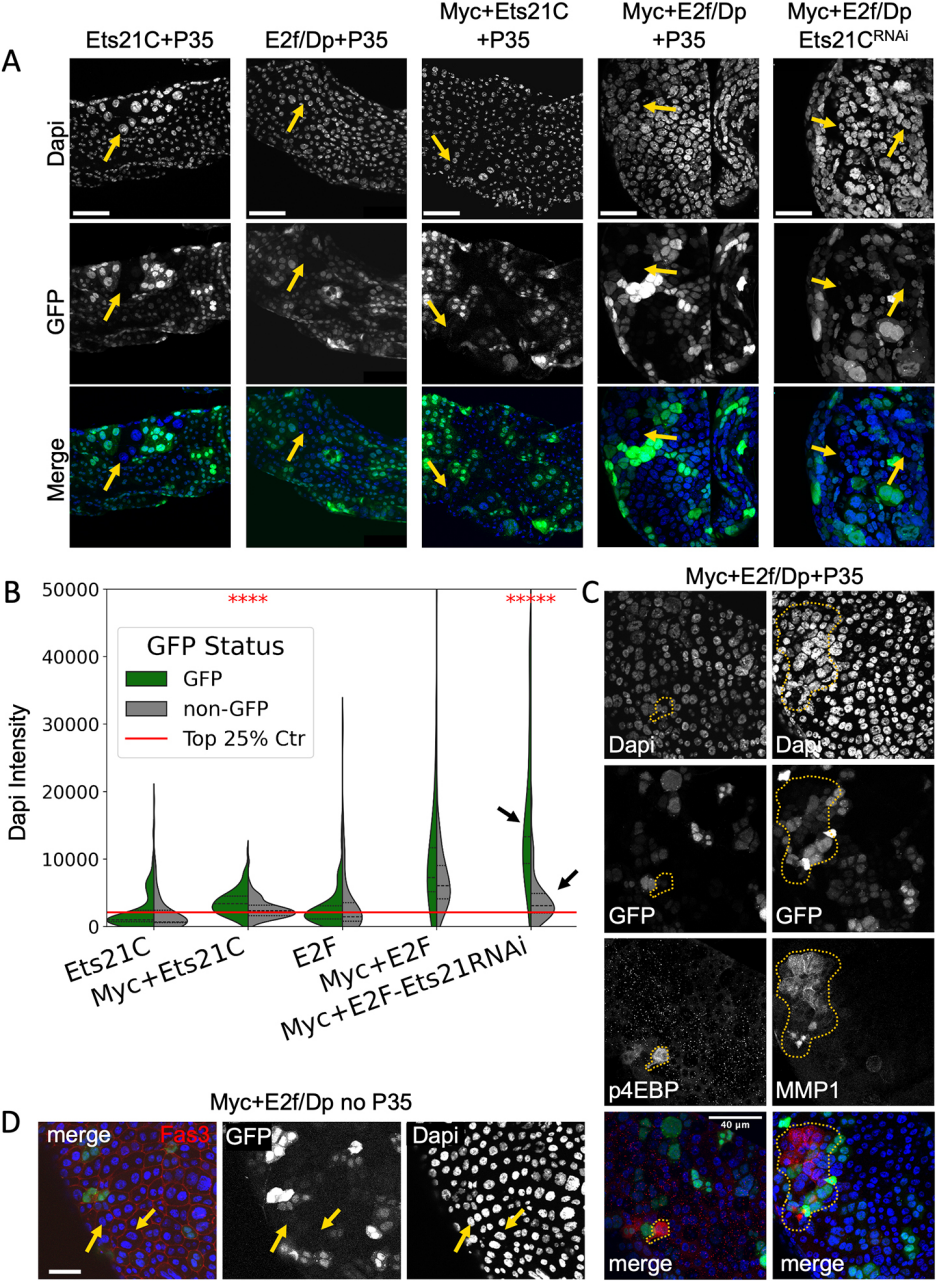
**Non-autonomous endocycling in GFP^−^ cells neighboring oncogene-expressing cells.** (A) Adult *Drosophila* accessory glands expressing Ets21C+P35, E2f/Dp+P35, Myc+Ets21C+P35, Myc+E2f/Dp+P35 and Myc+E2f/Dp+Ets21C^RNAi^ exhibit enlarged GFP^+^ nuclei as well as GFP^−^ enlarged nuclei near GFP^+^ cells (yellow arrows). Scale bars: 50 µm. (B) A split-violin plot of DAPI intensities for the indicated genotypes shown in A, showing GFP^+^ (green) versus GFP^−^ (gray) nearby cells. Black arrows indicate a significant change in DAPI intensity for GFP^+^ versus GFP^−^ nuclei when Ets21C^RNAi^ is co-expressed with Myc+E2f/Dp. For each genotype, 100 GFP^+^ and GFP^−^ nuclei were quantified across four biological replicates. Statistics used a mixed linear model regression model to account for within-replicate variability. The red line indicates the upper quartile of DAPI intensity for the control (expressing only P35 and GFP). ****P≤0.0001, *****P≤0.00001. (C) Examples of Mmp1 and p4EBP staining in GFP^+^ and GFP^−^ cells of Myc+E2f/Dp+P35-expressing glands. (D) Examples of GFP^+^ and GFP^−^ nuclei (yellow arrows) expressing Myc+E2f/Dp without P35. Sample size: *N*=4. Scale bar: 50 µm.

Throughout our studies, we noted frequent examples in tissues overexpressing Ets21C, as well as combinations of Myc+Ets21C and Myc+E2f/Dp, whereby GFP^−^ cells residing near GFP^+^ cells exhibited altered characteristics, suggesting that the oncogenic signaling in these conditions has non-autonomous effects. To assess this possibility, we examined nuclear DAPI intensity for 100 GFP^+^ cells and nearby GFP^−^ cells across four biological replicates for overexpression of Ets21C, Myc+Ets21C, E2f/Dp and Myc+E2f/Dp (Fig. 6A,B). We observed increased DAPI intensity across all genotypes for GFP^+^ cells. When we examined nuclear DAPI intensities for GFP^−^ cells near GFP^+^ cells, we also observed increased DNA content, suggesting non-autonomous induction of endocycling, which we also observed in our EdU labeling experiments. The non-autonomous effects of Myc+E2f/DP on nuclear size do not require P35, and removing P35 expression did not lead to detectable cell death as assayed by cleaved *Drosophila* Caspase 1 (also known as Dcp-1) (Fig. S10).

Non-autonomous effects of JNK signaling can be propagated through production of paracrine signaling factors, such as Upd ligands or diffusible reactive oxygen species (ROS), both of which can be triggered by aneuploidy (Clemente-Ruiz et al., 2016; Khan et al., 2017; La Fortezza et al., 2016; Muzzopappa et al., 2017; Santabarbara-Ruiz et al., 2015). Consistent with this, we observed non-autonomous activation of Mmp1 expression and p4EBP in adjacent GFP^−^ nuclei when Myc+E2f/Dp was expressed (Fig. 6C). Ets21C, which is induced by JNK, has also been shown to activate expression of ligands that have non autonomous effects on growth, such as *upd3*, *upd1*, *Mmp1* and *Pvf1* (Mundorf et al., 2019; Toggweiler et al., 2016). Therefore we examined the effect of knocking down Ets21C using RNAi in the presence of Myc+E2f/Dp co-expression. Knocking down Ets21C by RNAi with Myc+E2f/Dp co-expression had little effect on nuclear DAPI intensity in GFP^+^ cells but had a strong effect on reducing DAPI intensity in neighboring GFP^−^ cells (Fig. 6A,B). As the Myc+E2f/Dp+Ets21C RNAi line does not contain P35, we confirmed that the non-autonomous effect of Myc+E2f/Dp on GFP^−^ cells also occurs in the absence of P35 (Fig. 6D).

### Endocycling prostate cancer cells upregulate genes orthologous to those upregulated in oncogene-expressing *Drosophila* AGs

**Fig. 7.**
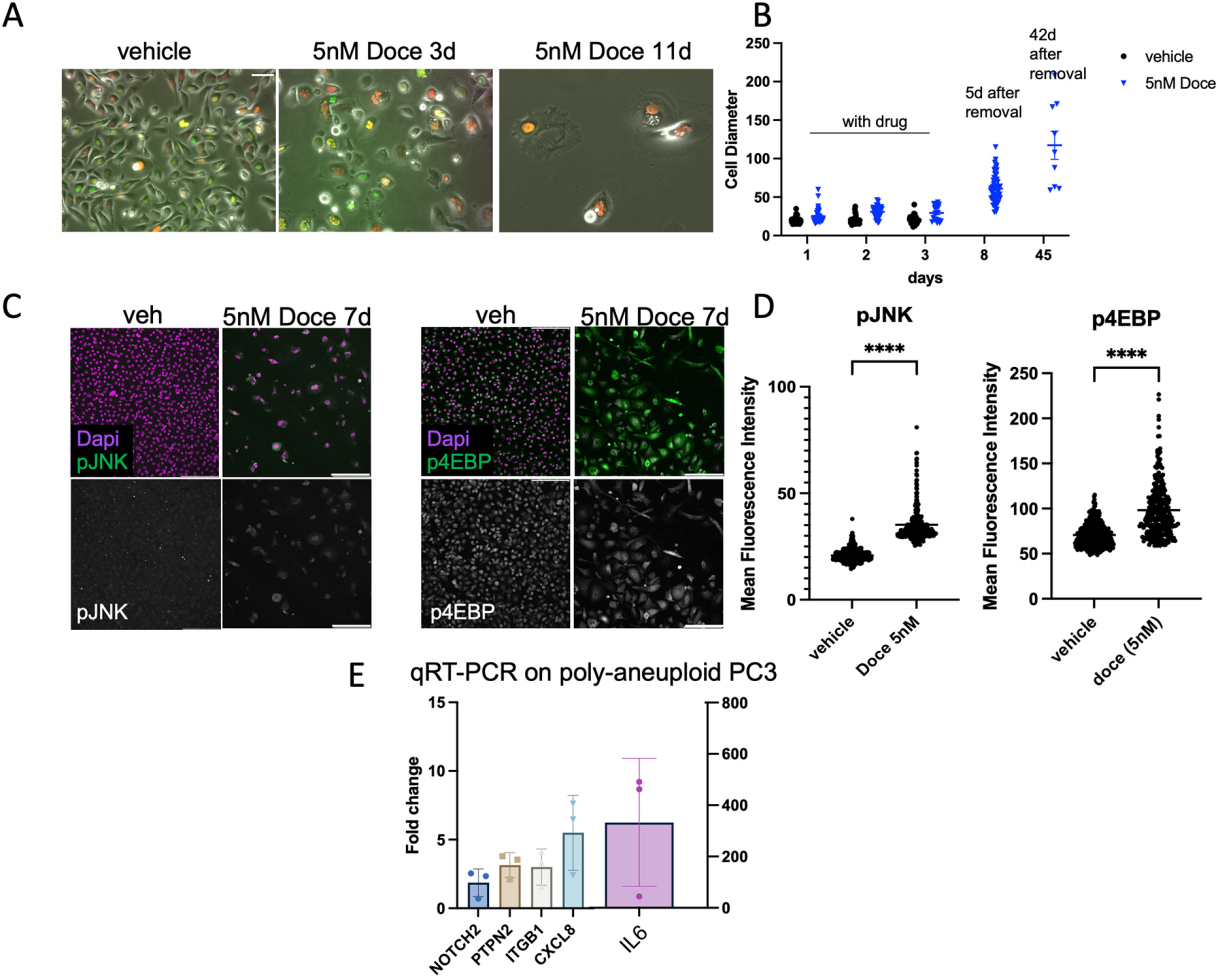
**Polyaneuploid prostate cancer cells upregulate stress and growth signaling genes orthologous to those upregulated in oncogene-expressing *Drosophila* accessory glands.** (A) Cultured PC3 cells stably expressing cell cycle reporters p27K-Venus (G0) and CDT1-mCherry (G0/G1) were treated with vehicle only (DMSO) or 5 nM docetaxel (Doce) for 3 days, after which most PC3 cells die. (B) After drug removal, long-term-surviving PC3 cells continue to grow for over a month as shown by increasing cell size. (C,D) PC3 polyaneuploid cells 5 days after drug removal exhibit increased pJNK and p4EBP. Methods for measurements of mean fluorescence intensity across three replicates with a two-tailed *t*-test with Welch's correction are described in the Materials and Methods. (E) qRT-PCR analysis of the indicated genes was performed on RNA isolated from surviving PC3 cells 10 days after drug removal. Gene expression levels were normalized across samples using *ACTB* with control RNA (from vehicle only-treated cells) set to 1 to indicate the fold change in expression. Note that fold change for *IL6* transcript is shown on the right *y*-axis, as average fold change exceeded 300. Scale bars: 50 µm (A), 200 µm (C).

After chemotherapy, a minority of cancer cells in the treated population exit mitosis and undergo cell cycle variants including endomitosis and endoreduplication. These cancer cells exhibit increased survival, hyperplasia and increased genomic content, i.e. they are in a large, polyaneuploid state (Kim et al., 2023; Lin et al., 2019; Pienta et al., 2021). These endocycling cells can also enter senescence-like states that can impact the tumor microenvironment in a non-autonomous manner (Lin et al., 2019; Niu et al., 2021). To examine whether oncogenic gene expression in *Drosophila* AG resembles the polyaneuploid state in human prostate cancer cells, we induced polyploidy in a PC3 human prostate cancer cell line using docetaxel treatment. By 3 days of culture with 5 nM docetaxel, most PC3 cells had died or arrested their cell cycle. Following our previously established protocols for endocycling cell formation (Kim et al., 2023; Mallin et al., 2023), we removed the docetaxel on day 3, provided cells with fresh culture medium and tracked continued cellular growth for up to 60 days in culture (Fig. 7A,B; Fig. S11). We confirmed that at least a portion of this growth is due to endocycling, evident from EdU incorporation in S-phase cells with enlarged and multiple nuclei (Fig. S11).

Our PC3 cell line is a clonal sub-line that stably expresses two cell cycle reporters, a p27-based Venus reporter that accumulates during the G0 phase of the cell cycle but is degraded during G1, S and G2/M (Oki et al., 2014), and a component of the FUCCI cell cycle reporter system, CDT1-N-terminus (1-32) fused to mCherry, which accumulates during G0 and G1 phases but is degraded during S-phase, G2 and M (Sakaue-Sawano et al., 2008). With these reporters, cell cycle arrest in G0 can be distinguished from cycling by live-cell imaging as well as flow cytometry (Pulianmackal et al., 2021). We found that most surviving cells after docetaxel treatment become quiescent by 21 days and remain in G0, with only a small fraction continuing to endocycle (Fig. S11).

The cell cycle arrest of surviving cells could be due to expression of cell cycle inhibitors in response to the endocycling state that results in increased genomic content. Indeed, we observed high levels of expression of the cell cycle inhibitor p21 (also known as CDKN1A), along with high levels of phosphorylated ATM, both of which are induced by DNA damage and genetic instability. We also observed a small number of endocycling cells with high p16 (also known as CDKN2A) expression, a cell cycle inhibitor that is normally epigenetically silenced in PC3 cells (Chi et al., 1997; Jarrard et al., 1997; Kondo et al., 2008), but may become expressed in a small subset of cells as they enter a senescence-like state in response to sustained DNA damage and cell cycle arrest (Steiner et al., 2000) (Fig. S12).

We next examined whether the surviving polyaneuploid PC3 cells upregulate the signaling pathways we observed in postmitotic AG cells. We observed upregulation of JNK (also known as JNK1-3 in human) signaling (via phosphorylation of JNK) and TOR signaling [via phosphorylation of 4EBP (also known as EIF4EBP in human)] in surviving PC3 cells (Fig. 7C,D). Based upon overlap with our RNAseq datasets and the wound-healing and *Ras^V12^,scrib^−/−^*-based tumor model datasets, we prepared a list of genes to examine for their expression in polyaneuploid PC3 cells. From a list of 35 *Drosophila* genes (which included genes within the TNF, EGF, PDGF, Notch, JAK/STAT and JNK pathways), we generated a list of ∼135 predicted human orthologs (Hu et al., 2011) (Table S3). We next examined data from docetaxel-treated PC3 cells accumulated from our other studies (Schmidt et al., 2025) to generate a shorter overlapping list of 13 genes, which we examined by quantitative reverse transcription PCR (qRT-PCR). We observed increased expression of several genes we saw upregulated in oncogene-expressing *Drosophila* glands by qRT-PCR in PC3 polyaneuploid cells, including *NOTCH2* (ortholog of *Notch*), *PTPN2* (ortholog of *Ptp61F*), *ITGB1* (ortholog of *Beta-integrin*), and the unpaired ligands *CXCL8* (encodes IL-8 protein) and *IL6* (Fig. 7E). Other genes from our ortholog list exhibited upregulated secretion by polyaneuploid PC3 cells, as measured by cytokine array on conditioned media from polyaneuploid PC3 cells at 10 days post treatment (Mallin et al., 2025). Secreted factors include several matrix metalloproteinases (orthologs of Mmp1), IL-6, TNF (Eiger ortholog), AREG and EGF (Vein, Spi, Grk orthologs) and PDGF-B (Pvf2 and 3 orthologs). These results suggest that our postmitotic model in the *Drosophila* AG recapitulates specific features of human prostate cancer driven by cells that survive chemotherapeutic treatment by entering the endocycling state.

## DISCUSSION

**Fig. 8.**
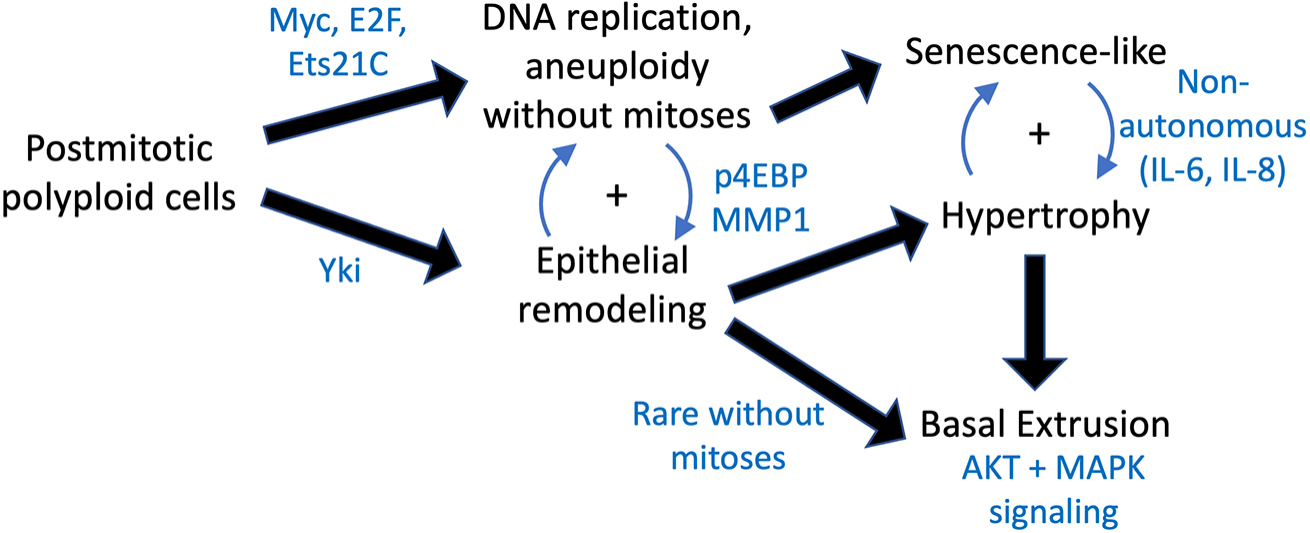
**A model for non-proliferative oncogenic signaling in the adult *Drosophila* accessory gland.** Oncogenic stimuli, such as activation of Myc, E2f or Ets21C, promote endocycling leading to aneuploidy, while oncogenic activation of Yki preferentially triggers epithelial remodeling. Both processes can engage each other, leading to extrusion or hypertrophy accompanied by gene expression resembling senescence-like states that can non-autonomously promote tissue hypertrophy.

We present an *in vivo* postmitotic and polyploid prostate cancer model in the *Drosophila* AG. Our model displays many phenotypic hallmarks of tumorigenesis, such as disruption of epithelial organization, apical invasion and basal extrusion, cellular and nuclear hypertrophy, and compromised tissue differentiation. All of these phenotypes could be activated in postmitotic cells in the absence of proliferation. Our results reveal that postmitotic cells can activate a conserved network of tumorigenesis and wound-healing-related genes, and likely engage in recently described feedback loops between a JNK-dependent senescent-like gene expression state and a JAK/STAT-induced pro-growth state that engages with the tissue microenvironment in an autocrine as well as paracrine manner (Jaiswal et al., 2023). Our results demonstrate that these feedback loops and gene expression programs are cell states that can be achieved entirely independent of cell proliferation and can even, under rare conditions, result in basal extrusion phenotypes reminiscent of neoplasia without proliferation. Finally, our investigations into prostate cancer cells that survive therapeutic insult by engaging an endocycling program suggest that these signaling states and gene expression changes are conserved in non-mitotic polyploid human prostate cancer cells. Fig. 8 presents a model for non-proliferative oncogenic signaling in the adult *Drosophila* accessory gland, illustrating that oncogenic pathways can drive epithelial remodeling and tumorigenic features without inducing proliferation.

### Non-proliferative polyploid cells in cancer

Not all cells in tumors proliferate. Polyploid cancer cells are frequently found in human cancerous tissues, with ∼37% of all human tumors reported in The Cancer Genome Atlas Pan-Cancer dataset showing evidence of polyploid DNA content (Bhathal et al., 1985; Liu, 2018). Polyploidy has been found to occur via several mechanisms, including increasing the number of nuclei, cytokinesis failure (Caldwell et al., 2007; Fujiwara et al., 2005; Meraldi et al., 2002), cell cannibalism by entosis (Augimeri et al., 2023; Krajcovic and Overholtzer, 2012) and cell fusion (Brodbeck and Anderson, 2009; Hosaka et al., 2004). Additionally, cells can become polypoid by increasing genomic content via endoreplication (Holland and Cleveland, 2009).

Polyploid giant cancer cells have been associated with resistance to cancer therapy in cell culture experiments (Barok et al., 2011; Kim et al., 2023; Lin et al., 2019; Liu, 2018), and can be induced by factors such as hypoxic conditions, chemotherapeutic drugs and irradiation treatments *in vitro* (Fei et al., 2019; Kaur et al., 2015; Lanvin et al., 2013), which can result in increased migration and invasion capabilities, epithelial–mesenchymal transition-related protein expression and MMP expression (Fei et al., 2019; Lv et al., 2014; Mallin et al., 2023; Wang et al., 2019; Zhou et al., 2022).

Our cell culture model for endocycling cancer cells is limited in that it is a monoculture. The chemotherapy-induced endocycling prostate cancer cells exhibit JNK activation, a DNA damage response, and gene expression changes similar to those observed in the *Drosophila* AG after oncogenic activation. Most strikingly, we observed very strong upregulation of IL6 and CXCL8 expression, consistent with senescence-like phenotypes in the polyaneuploid state (Niu et al., 2021; Pienta et al., 2022, 2021). Interestingly, several STATs (STAT3, STAT5A/B) are genetically mutated and lost in PC3 cells (Seim et al., 2017; Spiotto and Chung, 2000). This suggests that the non-autonomous proliferative effects of the surviving large, polyaneuploid PC3 cells, perhaps through IL-6 and IL-8 release, should be examined in co-culture experiments with other prostate cell lines that retain the full JAK/STAT signaling pathway. This will also be important to examine in co-culture studies with immune cells and other cells of the prostate cancer microenvironment. PC3 cells have been shown to potentially signal via IL-6 protein in a paracrine manner (Lou et al., 2000). Future work should confirm the secretion of these paracrine factors by the cells in the large polyaneuploid state and examine their effects on other cells non-autonomously, to gain a deeper understanding of how cancer cells in this state impact the tumor microenvironment.

### The timing of oncogene activation and cell cycle exit

Activation of oncogenic Ras^V12^ expression during the proliferative phase of *Drosophila* AG development was shown to frequently lead to neoplastic-like basal extrusions of cells that lack cell polarity and exhibit signaling consistent with a tumorigenic state (Rambur et al., 2020). In our study, we find that activation of oncogenic Ras^V12^ expression in the adult *Drosophila* AG rarely progresses to basal extrusions, despite high levels of MAPK and PI3K/Akt signaling. We attribute this to loss of cell cycle and cell state plasticity after cell cycle exit and maturation in the adult gland. The *Drosophila* AG cells exit from the mitotic cell cycle ∼55-60 h after puparium formation, but it is not known exactly when the AG cells commit to their terminal cell fate. After exit from the mitotic cell cycle, the AG undergoes additional endocycles, which appear to also occur in the Ras^V12^-induced basal extrusions, suggesting that cell fate is not totally compromised, although other aspects of epithelial differentiation, such as cell polarity, may be abrogated. We recently showed that the AG main cells exit from the cell cycle ∼4-6 h post eclosion in adults, after an additional round of endocycling, with only rare cells (<2% of the gland) continuing to endocycle (Box et al., 2024). As our genetic manipulations in this study occur after exit from the endocycle, we speculate that the additional developmental time somehow reshapes and limits the AG response to Ras^V12^ overexpression. Studies comparing the gene expression networks induced by Ras^V12^ at different developmental times in this tissue could reveal the molecular basis for these differential effects of oncogenic activity.

Although basal extrusions are rare in our study, we readily observed apical protrusions and invasions into the luminal space of the AG. We also observed loss of proper cell–cell junctional protein localization, suggesting that partial defects in cell polarity occur, although not to the extent observed in basal extrusions (Rambur et al., 2020). Epithelial polarity disruptions can lead to the aberrant localization of growth factor receptors, potentially creating feedback loops that promote tumorigenic signaling and impact cells non-autonomously. Additionally, the upregulation of Mmp1, observed in our models, is indicative of JNK signaling, cellular stress and active invasion into surrounding tissues. In cancer, even partial polarity loss can be associated with the acquisition of invasive capabilities, often marked by the enhanced expression of MMPs. Of note, recent work has also suggested that inhibition of Yki activity in the adult AG can lead to some of the phenotypes we report here under Yki activation, including Mmp1 expression, cellular hypertrophy, epithelial disruption and growth signaling (Bhattacharya et al., 2024). This suggests that Yki activity may need to be carefully controlled as Yki activity levels that are too high or too low could induce stress responses in this tissue.

In the proliferative developing fly eye, neoplastic-like conditions have been shown to generate polyploid cells that promote tumor growth. In these cells, JNK signaling and Yki activity cooperate to promote growth while levels of the mitotic Cyclin, Cyclin B are suppressed, thereby preventing entry into mitosis leading to endoreplication (Cong et al., 2018). However, it is unclear in this developmental context whether Cyclin B suppression occurs at a transcriptional or post-transcriptional level. Although we see mRNA for several mitotic regulators increase in the Myc+E2f/Dp-expressing AGs (e.g. *aurA*, *aurB*, *Cdk1*, *CycA*, *polo*), *Cyclin B* transcript remains low, suggesting that it may be transcriptionally silenced in the postmitotic gland. An important difference in our model compared to the developing fly eye neoplastic-like tumors is that the cells undergoing hypertrophy or extrusion in the adult AG are not already in a proliferative state. Further work will be needed to tease apart whether cycling versus postmitotic endocycling cells engage different mechanisms to limit mitoses and induce polyploidy.

### JNK signaling, Ets21C induction and non-autonomous effects in the absence of proliferation

In the context of Myc+E2f/Dp, Myc+Ets21C, Ets21C and E2f/Dp overexpression, evidence of excessive endoreplication and aneuploidy was observed, indicative of genomic instability. Aneuploidy can lead to proteotoxic stress due to imbalances in gene expression that result in JNK activation, ROS production, dysregulation of autophagy and, ultimately, hallmarks of tumorigenesis (Joy et al., 2024). Our RNAseq data suggest that this also occurs in postmitotic cells of the AG, as we observed upregulation of genes involved in DNA damage, genetic instability, response to ROS and protein misfolding, although, unlike the aneuploidy arising from chromosomal instability in proliferating tissues, the aneuploidy in endocycling AGs likely arises from incomplete S-phases. Our gene expression profiles indicate strong upregulation of JNK signaling, with activation of known targets such as *puckered*, *Ets21C* and *Mmp1*. In addition, recent work has revealed non-autonomous signaling induced by a senescence-like program downstream of JNK signaling (Jaiswal et al., 2023) that can also be activated in non-dividing polyploid cells under pro-growth conditions (Huang et al., 2024).

In *Drosophila*, Ets21C is upregulated in response to JNK signaling (Kulshammer et al., 2015; Toggweiler et al., 2016) as well as inactivation of the transcriptional repressor *capicua* (*cic*) (Jin et al., 2015). Here, we show that JNK signaling is increased in prostate cancer polyaneuploid cells, and human CIC has been shown to be a tumor suppressor in prostate cancer (Gupta et al., 2022; Seim et al., 2017), suggesting that the *Drosophila* AG recapitulates key signaling features of the human cancer, despite the postmitotic state of the fly prostate-like cells. Cic function is downregulated by Ras/MAPK signaling, through degradation, nuclear export or both (Astigarraga et al., 2007; Grimm et al., 2012; Jin et al., 2015; Roch et al., 2002; Tseng et al., 2007). Although we do not observe MAPK activity via phosphorylated ERK in our hypertrophic AGs, we do find high phosphorylated ERK in cyst-like basal extrusions. The JNK signaling induced in AGs expressing oncogenes may promote Ets21C activation, while the additional MAPK activation observed in basal extrusions may further elevate Ets21C via Cic inactivation to drive cell cycle targets to induce endocycling. Both Ets21C and Cic directly impact cell cycle targets involved in endoreplication and DNA synthesis, with Cic acting as a repressor and Ets21C acting as an activator (Jin et al., 2015; Zhang et al., 2022). These pathways converge on genes that promote endocycling activated by Myc and E2f/Dp, potentially leading to the excessive endocycling and nuclear anaplasia we observe in Myc+E2f/Dp conditions. The upregulation of *Drosophila Ets21C* in the AG in response to oncogenic signaling could be particularly significant, considering its homology to the human *ERG* gene and other ETS transcription factors, known for their involvement in prostate cancer (Adamo and Ladomery, 2016; Nicholas et al., 2019; Qian et al., 2022; Tomlins et al., 2005). Our data suggest that Ets21C plays roles in promoting endocycling and cell growth both cell autonomously and non-autonomously, consistent with studies revealing paracrine targets of Ets21C in proliferative and wound-healing contexts (Mundorf et al., 2019; Toggweiler et al., 2016; Worley et al., 2022). Our *Drosophila* model offers an opportunity to further examine the role of Ets21C in promoting tumorigenic signaling in non-mitotic cells and suggest a role for ERG in polyploid cells of prostate tumors.

### A common tumor gene expression profile occurs even in the absence of proliferation

Our transcriptomic analysis of the response to Myc+E2f/Dp and Yki*+Dp in the *Drosophila* AG revealed gene expression changes that overlap significantly (10.97-fold greater than expected by chance) with a common wound healing and tumorigenesis gene expression program (Floc'hlay et al., 2023). Interestingly, our results suggest shared commonality in the regulatory networks of tumor behavior and response to wounding in *Drosophila* AG and human prostate cancer cells, even in the absence of cellular proliferation (MacCarthy-Morrogh and Martin, 2020). This work is the beginning of disentangling the contribution of cell cycle plasticity to tumorigenic gene expression networks, and further studies in tissues with variant cell cycles could reveal additional aspects of tumorigenic programs that are proliferation independent.

## MATERIALS AND METHODS

### Staining and fixation

*Drosophila melanogaster* samples and dissected male AGs were fixed in a 4% paraformaldehyde/1× PBS solution for 30 min, followed by three 10 min washes in 1× PBS with 0.1% Triton X-100 (PBST). The samples were then permeabilized with 0.5% Triton X in PBS for 30 min and blocked in a solution of PBS, 0.1% Triton X-100 and 1% bovine serum albumin (BSA) for 10 min. Primary antibody incubation was conducted in PBS, 0.1% Triton X-100 and 1% BSA at 4°C overnight. This was followed by three washes in PBST and incubation in secondary antibodies conjugated with the required fluorophores, mixed in PBS with 0.3% Triton X-100, 0.1% BSA and 2% normal goat serum (PBT-X+2%NGS) at room temperature for 4 h or overnight at 4°C. For nuclear staining, DAPI was applied 1:1000 in PBST for 10 min, followed by three rinses of PBST, for 10 min each. The samples were mounted on glass slides using Vectashield mounting medium. Imaging was performed using a Leica SP5 confocal microscope.

Primary antibodies used in this study included Phospho Histone H3 Ser10 Mitosis Marker mouse (1:500; Millipore, 06-570), MMP1 catalytic domain mouse [1:10; Developmental Studies Hybridoma Bank (DSHB), 5H7B11-s, 3B8D12-s, 3A6B4-s], Dcp1 Cleaved *Drosophila* rabbit (1:1000; Cell Signaling Technology, 9578s Asp216), Integrin Beta PS (Mys) (1:100; DSHB, CF.611), Discs Large mouse (1:500; DSHB, 4F3), dp-ERK (1:500; Cell Signaling Technology, 4370s), Fas III (1:500; DSBH, 7G10), Coracle-heavy (1:100; DSHB, C615.16-s), P-AKT (1:200; Cell Signaling Technology, 4054s) and P-4E-BP (1:200; Cell Signaling Technology, 2855S). Secondary antibodies used in this study were Alexa Fluor 568 goat anti-mouse (1:2000; Invitrogen, A11031) or Alexa Fluor 568 goat anti-rabbit (1:2000; Invitrogen, A-11011), Alexa Fluor 594 F(ab′)2 goat anti-mouse (1:2000; Invitrogen, A11020) and Alexa Fluor 594 F(ab′)2 goat anti-rabbit (1:2000; Invitrogen, A11072).

### EdU labeling

EdU labeling was performed as instructed by the manufacturer's protocol using Click-iT™ Plus EdU Cell Proliferation Kit for Imaging, Alexa Fluor™ 555 dye (Thermo Fisher Scientific, C10638). For EdU assays, male flies were exposed to a 37°C water bath for 20 min on the day of eclosion. Animals were housed in a 25°C incubator and aged. Male flies were aged for 6 days post eclosion before being fed 1 mM EdU in 10% sucrose with blue food coloring for 4 days. A mixture of EdU and sucrose was applied to Whatman paper, which was then placed inside empty vials. This setup was refreshed every 2 days to prevent contamination.

### Fly rearing and mating

#### Fly stocks

Fly stocks (prefixed with ‘BL’ if from Bloomington *Drosophila* Stock Center) used were as follows: *w/w; UAS-P35/Cyo-GFP; act>CD2>Gal4, UAS-GFP(nls)/TM3-Ser-GFP* (*UAS-P35* from BL#5072, *act>CD2>Gal4* from BL#4780); *y,w,hs-flp^12^; +; UAS-yki* (*yki* from BL#28819); y,w,hs-flp^12^; UAS-E2f, UAS-DP/Cyo-GFP; yki* [*UAS-E2f1, UAS-DP* from Neufeld et al. (1998), constitutively active UAS-Yki* from BL#28817]; *y,w,hs-flp^12^; UAS-E2f, UAS-DP/Cyo-GFP; UAS-dMyc42/TM6B* [*UAS-E2f1, UAS-DP* from Neufeld et al. (1998), *dMyc* from BL#9675]; *y,w,hs-flp^12^;+;UAS-E2f1, UAS-Dp/TM6B* (BL#4770); y,w,hs-flp^12^; +; +; *y,w,hs-flp^12^; UAS-Rasv12/ CyO-GFP; +* (BL#5788 or BL#64196); *y,w,hs-flp^12^; +; UAS-dMyc/TM6B* (*dMyc* from BL#9675); *w/w; UAS-Ets21C/Cyo-GFP; act>CD2>Gal4, UAS-GFP(nls)/TM6B* [*Ets21C* from Külshammer et al. (2015), *act>CD2>Gal4* from BL#4780]; and *w/w; UAS-Ets21C-RNAi/Cyo-GFP; act>CD2>Gal4, UAS-GFP(nls)/TM6B* (*Ets21C-RNAi* Vienna *Drosophila* Resource Center #106153, *act>CD2>Gal4* from BL#4780).

For antibody staining, male flies 1-3 days after eclosion had transgene expression induced by exposing animals to a 37°C water bath for 20 min. Animals were housed at room temperature and aged for various timepoints. Sample sizes were not pre-determined. In figure legends, ‘*N*’ indicates biological replicates from individual animals.

We used FlyBase (release FB2025_01) to find information on phenotypes, gene function, *Drosophila* stocks and gene expression (Ozturk-Colak et al., 2024).

### Imaging/measurements/DAPI quantifications

Imaging was conducted using a Leica SP5 confocal microscope at various magnifications, with a *z*-section thickness of 1 μm. Maximum-intensity projections were created using LAS AF software (Leica Microsystems). Image quantification was carried out with Fiji software. For this process, regions of interest (ROIs) of similar size were manually outlined based on nuclear (DAPI) staining or antibody staining. To normalize the area for quantification, the integrated density was adjusted by subtracting the background measured in ROIs outside the tissue. Data for nuclear intensity and line scans are available in Tables S1 and S2.

### Flow cytometry

We followed the AG nuclear isolation and flow cytometry from Box et al. (2024) for ovaries and AGs. Vybrant DyeCycle Violet stain (Invitrogen) at 1:1000 was used for nuclear staining. Samples were lightly vortexed just prior to running on an Attune Flow Cytometer (Thermo Fisher Scientific).

### Bulk RNAseq

Male flies, 10 days post eclosion, were selected for dissection after visually confirming high transgene/GFP expression. Their AGs were extracted and immediately placed in TRIzol (Invitrogen, 15596026) for RNA preservation. Following the manufacturer's instructions, we isolated RNA from these samples. The prepared RNA samples were then sent to the University of Michigan's Sequencing Core for further analysis.

At the sequencing facility, we employed PolyA selection to create barcoded libraries for each sample. The integrity and quality of these libraries were verified using a Bioanalyzer and quantitative PCR. We conducted sequencing on the NovaSeq SP 100 cycle platform, ensuring high-read quality through FastQC assessments.

For data analysis, sequences were aligned using the Sleuth/Kallisto pipeline, with reference to the *Drosophila melanogaster* genes based on the Ensembl BDGP6.28 database. Differentially expressed genes were identified based on a log2 fold change threshold of ±1 (equivalent to a 2-fold change) and an adjusted *P*-value less than 0.05, unless otherwise stated.

### Cell culture

PC3 cells (ATCC) expressing G0-Venus and G1-mCherry fluorescent reporters were cultured as described previously (Pulianmackal et al., 2021). This line was authenticated and tested for mycoplasma as described (Zielske et al., 2023). For induction of polyploidy, cells were seeded into 12-well plates or chamber slides at a density of 1-5 million cells/ml and treated with vehicle [dimethyl sulfoxide (DMSO)] or 5-10 nM docetaxel (Cayman Chemical) in DMSO for 72 h. Medium containing vehicle or docetaxel was removed, adhering cells were washed with 1× PBS, fresh medium without DMSO or docetaxel was added, and cells were cultured for the indicated number of days post drug removal. For EdU labeling, 10 µM EdU was added to the medium for 3 h or 20 h prior to cell fixation in 4% PFA/PBS. EdU incorporation was assayed using an Alexa Fluor 647 Click-IT™ EdU detection kit (Thermo Fisher Scientific, C10640).

Phosphorylated JNK (pJNK) and p4EBP signal was quantified in PC3 cells using ImageJ. Briefly, confocal images of cells were used to create a cell mask by imposing a threshold (set to the same level as vehicle controls) with Gaussian blur of 1.5 radius to generate outlines of cells for the ROI manager, which was applied to the channel for the staining of interest to quantify mean fluorescence intensity via ‘measure’. Size limitations were imposed in the ‘Analyze particles’ function (pixels 10-5000) to remove particles smaller than 50% of a diploid cell but retain large polyploid cells. Values were plotted using GraphPad Prism, and a *t*-test with Welch's correction was performed.

### qRT-PCR

For qRT-PCR assays, total RNA was isolated from vehicle-treated PC3 or docetaxel-treated PC3 cells 10 days after drug removal. Reverse transcription with polyT and random primers was performed to generate cDNA, which was subsequently used for qRT-PCR. Three biological replicates were performed. Primer sequences for qRT-PCR assays are provided in Table S4.

### Statistical analysis and visualization

Our data were analyzed, statistically tested and visualized using a suite of Python packages, including Pandas, NumPy, Matplotlib, Matplotlib_venn, Seaborn, Networkx, Scipy and Statsmodels.api. Additionally, we employed Attune and LAS AF software, along with the R package Sleuth, for assessing differential expression and calculating fold changes.

For the EdU comparisons (Fig. 5), we applied the two-sided Wilcoxon–Mann–Whitney test using SciPy to compare experimental genotypes against the control condition. In analyzing nuclear intensity (Fig. 6), we leveraged statsmodels.api to conduct mixed linear model regression, comparing our experimental genotypes with the control. This dataset included 200 observations across four biological replicates per condition, with the mixed linear model regression offering a robust framework to account for within-replicate variability, unlike the Wilcoxon–Mann–Whitney test.

Line-scan data (Fig. 2) generated from Fiji were analyzed using NumPy for quartile range calculations and Matplotlib for visualization. We employed the Wilcoxon–Mann–Whitney test for violin plots to compare GFP^+^ nuclear intensity against non-GFP nuclear intensity (Fig. 6). Venn diagrams were created with Matplotlib and Matplotlib_venn (Fig. 4).

For constructing node graphs (Fig. 4), we used Networkx and Matplotlib, focusing on genes with an adjusted *P*-value of 0.05 or lower and a fold change of ±1 or greater. Gene Ontology (GO) term analysis of differentially expressed genes, adhering to the same thresholds, was conducted using the National Institutes of Health (NIH)'s Database for Annotation, Visualization, and Integrated Discovery (DAVID) for biological process categorization. Only GO terms meeting these significance and fold-change criteria were selected.

When creating heatmaps, we utilized GO datasets from Amigo 2, with differential gene expression thresholds for adjusted *P*-value and fold change as outlined for each genotype.

### Fold enrichment and hypergeometric distribution

Fold enrichment calculations provide a ratio comparing observed frequency in the subset to expected frequency if the distributions were random. Hypergeometric calculations use the hypergeometric distribution to calculate the probability of observing overlap between two or more sets, considering the sizes of the sets and the total population. We compared the upregulated gene sets for fold enrichment and statistical significance via hypergeometric calculations. We used a threshold of log_2_ fold change of 1 (2-fold change or greater) and an adjusted *P*-value of 0.05 or smaller for the bulk RNAseq data we generated. We used genes identified as statistically significant and upregulated in published work (listed in Table S5). The Python package Scipy.stats was used to calculate the hypergeometric probability number and the math package for converting the fold change to log_2_. We compared our datasets to the datasets in Table S5.

## Supplementary Material

10.1242/dmm.052001_sup1 Supplementary information

Table S1. Line plot data of Coracle fluorescence intensity in accessory gland epithelium (Fig. 3).

Table S2. Quantification of nuclear intensity in GFP+ vs non-GFP+ cells from *Drosophila* accessory glands (Fig. 6).

Table S3. Comparative gene expression profiles: *Drosophila* prostate-like endocycling cells vs polyaneuploid cancer cells.
