# Supplementary material for: Oncogenic signaling in the Drosophila prostate-like accessory gland activates a pro-tumorigenic program in the absence of proliferation
Source: Dis Model Mech. 2025 Apr 30;18(4):dmm052001. doi: 10.1242/dmm.052001 (PMC12067084; doi:10.1242/dmm.052001)
Supplement: Supplementary information [file dmm-18-052001-s1.pdf]

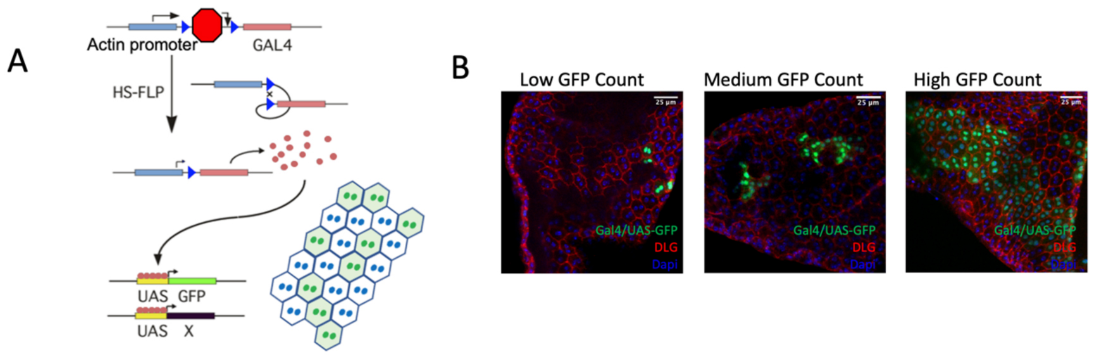

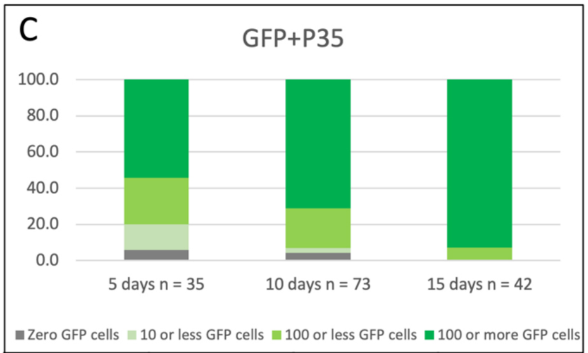

Y,W,HS	5 days n = 35	10 days n = 73	15 days n = 42
Zero GFP cells	5.7	4.1	0.0
10 or less GFP cells	14.3	2.7	0.0
100 or less GFP cells	25.7	21.9	7.1
100 or more GFP cells	54.3	71.2	92.9

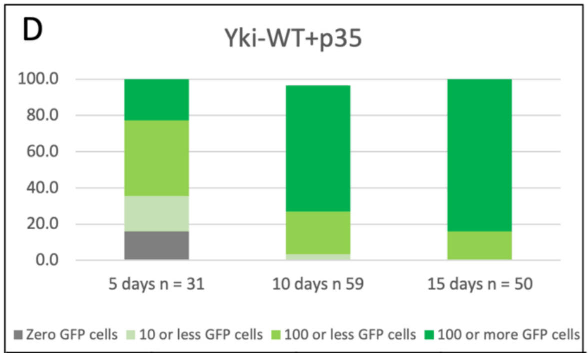

Yki-WT	5 days n = 35	10 days n = 73	15 days n = 42
Zero GFP cells	16.1	0.0	0.0
10 or less GFP cells	19.4	3.4	0.0
100 or less GFP cells	41.9	23.7	16.0
100 or more GFP cells	22.6	69.5	84.0

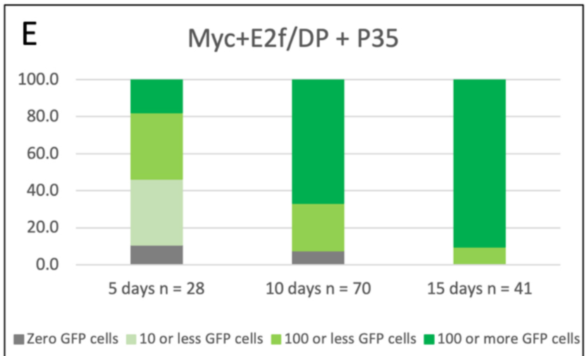

Myc+E2f,Dp	5 days n = 28	10 days n = 70	15 days n = 41
Zero GFP cells	10.7	7.7	0.0
10 or less GFP cells	35.7	0.0	0.0
100 or less GFP cells	35.7	25.6	9.8
100 or more GFP cells	17.9	66.7	90.2

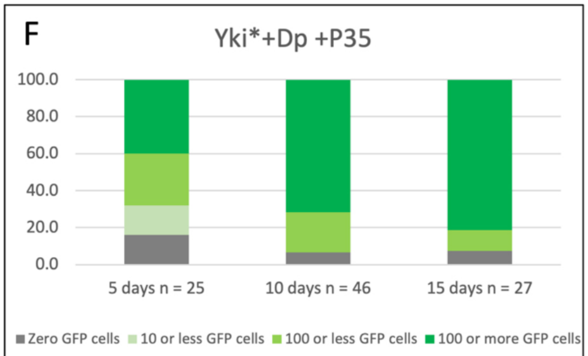

Yki*+Dp	5 days n = 28	10 days n = 70	15 days n = 41
Zero GFP cells	16.0	6.5	7.4
10 or less GFP cells	16.0	0.0	0.0
100 or less GFP cells	28.0	21.7	11.1
100 or more GFP cells	40.0	71.7	81.5

Fig. S1. Heat-shock parameters and Gal4/UAS-GFP expression for adult accessory gland. A. Cartoon diagram of the "flipout" Gal4/UAS system used to induce postmitotic expression. Hexagonal cells of the gland are represented with green shading for GFP expression and blue nuclei. B. Examples of the level of flipout and GFP expression in glands after 20 minutes heatshock at 1-2d post eclosion. C. Summary data of transgene expression levels after 20 minutes heatshock at 1-2d post eclosion and aging for 5-15 days. Note the level of flipout varies based on genotype despite all genotypes using the same hs-flp transgene on the X chromosome (hs-flp[12]).

Disease Models & Mechanisms • Supplementary information

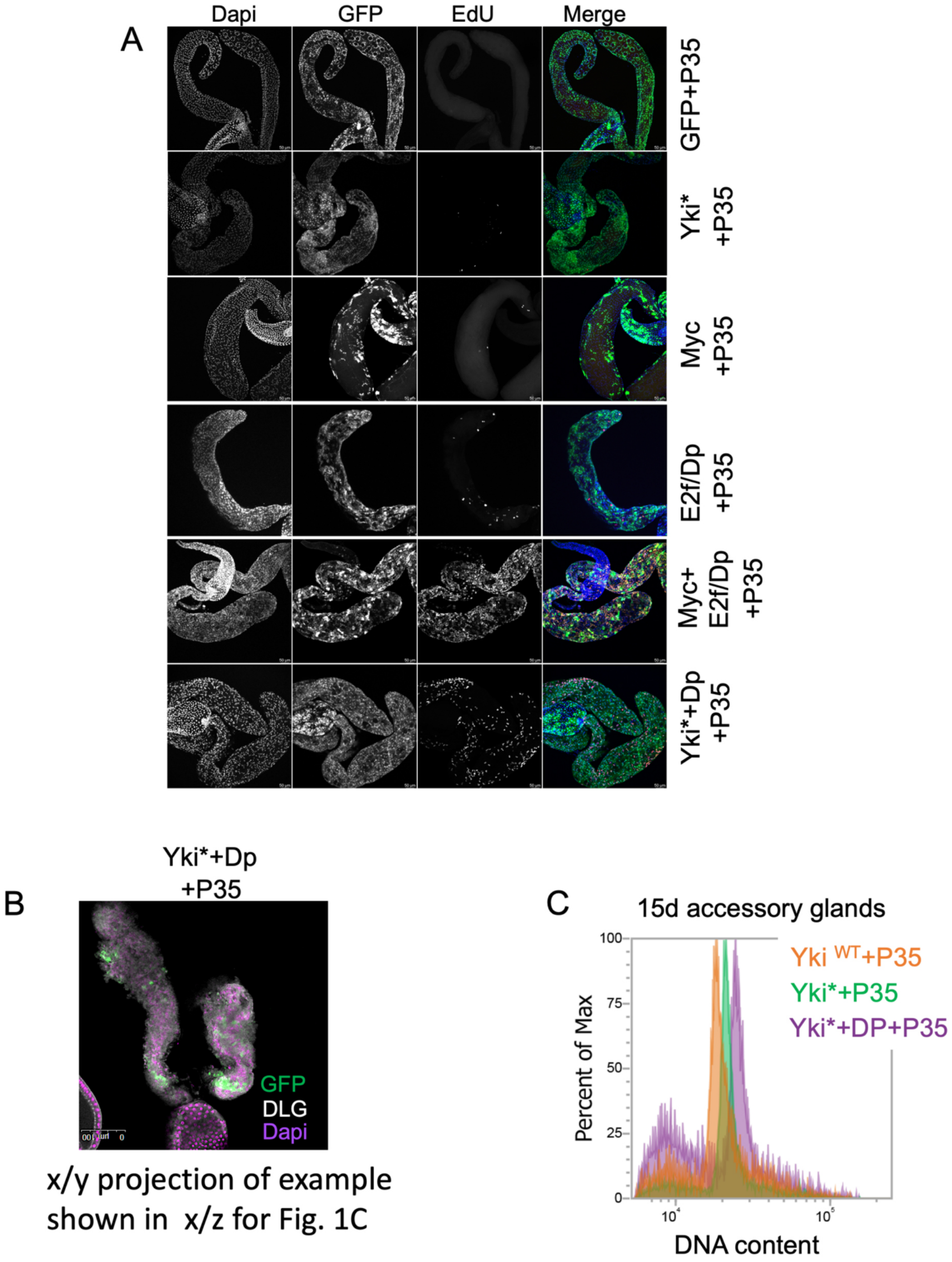

Disease Models & Mechanisms • Supplementary information

Fig. S2. S-phases and EdU labeling in accessory glands.
A. The indicated transgenes were induced by 20 min. heatshock at 1-2d post eclosion. Flies were fed EdU from day 6-10 and stained on day 10. B. The x/y confocal projection for the Yki*+Dp+P35 tissue from Fig. 1C is shown. This view shows that the cells have invaded the lumen causing the overall gland size to shrink. C. Flow cytometry to measure DNA content of gland nuclei expressing the indicated transgenes for 15d.

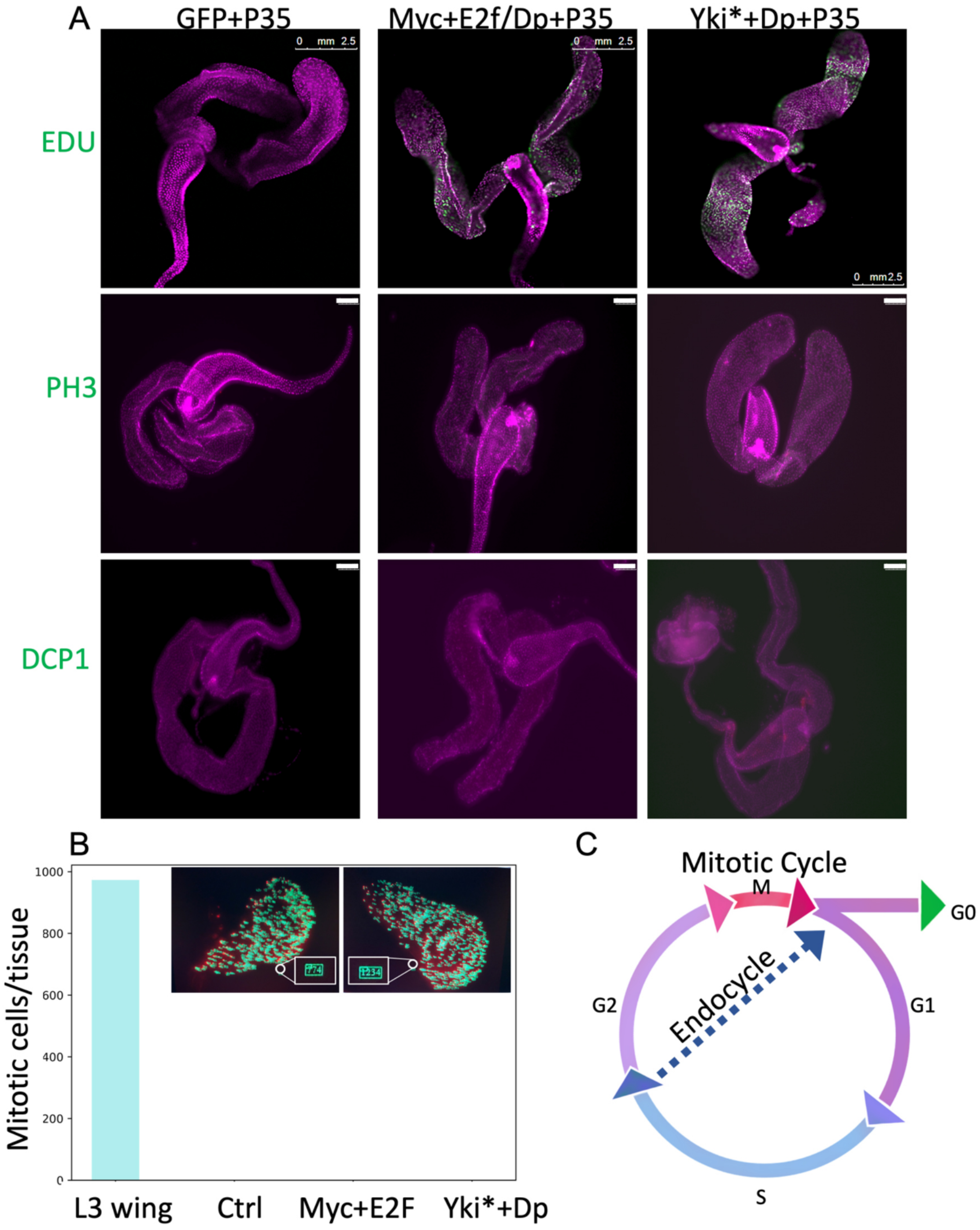

Disease Models & Mechanisms • Supplementary information

Fig. S3. Endoreplication occurs without mitoses or apoptosis in accessory glands.

A) The indicated transgenes were induced at 1-2d post eclosion. Flies were fed EdU for 24h and stained on day 5 for EdU, mitotic marker phosphohistone H3 (PH3), and cell death marker DCP-1. Scale bar in the PH3 and DCP-1panels is 100µm. Larval wing discs were co-stained in the tube with each accessory gland sample to ensure the mitotic marker labeling was working. B. PH3 quantifications show no PH3 positivity in glands, but extensive mitoses in wings. An example of mitotic index measurement for wings via segmentation of mitotic figures in Image J is shown in the inset. C) Cartoon diagram of the endocycle which skips mitosis to increase nuclear DNA content.

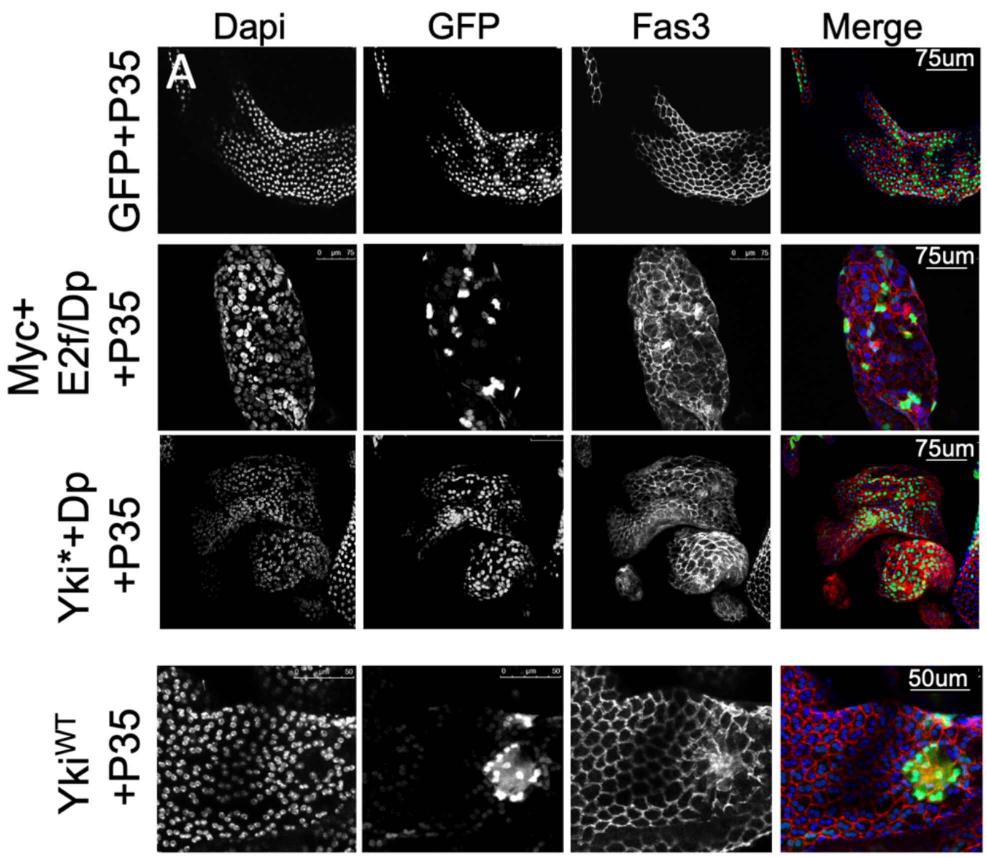

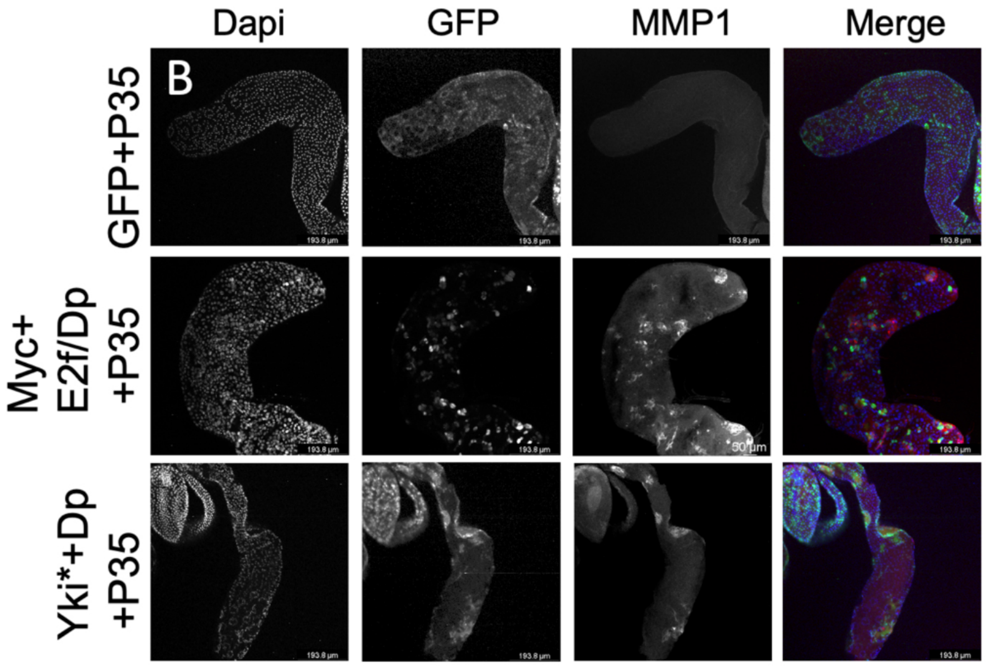

Fig. S4. Epithelial remodeling in oncogene expressing accessory glands.
A) Adult glands expressing the indicated oncogenic transgenes at 10d show Fas III mis localization. Sample sizes: GFP+P35 N = 5, Myc+E2f/Dp+P35 N = 4, Yki*+DP+P35 N = 6. B) Adult glands expressing the indicated oncogenic transgenes at 20d show Matrix Metalloproteinase 1 (MMP1) upregulation. Sample size: GFP+P35 N=12, Myc+E2f/Dp+P35 N=12, Yki*+Dp+P35 N= 13.

Disease Models & Mechanisms • Supplementary information

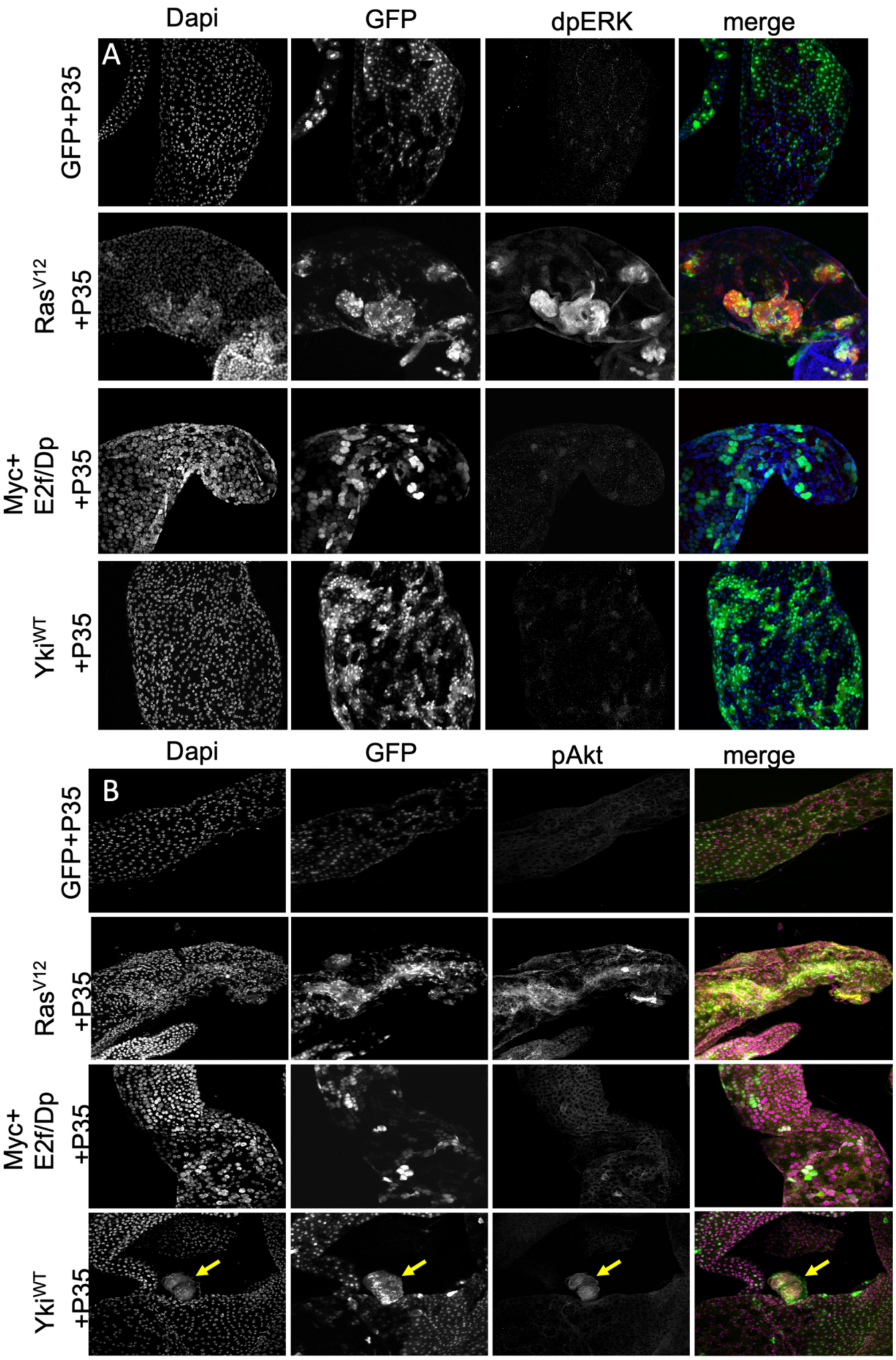

Disease Models & Mechanisms • Supplementary information

Fig. S5. MAPK and PI3K/AKT signaling in oncogene-expressing glands.
A) Accessory glands overexpressing the indicated oncogenic transgenes at 5-20d were stained for markers of MAPKinase signaling (di-phospho-ERK dp-ERK) and (B) PI3K/AKT signaling (phospho-AKT). Ras^{V12} expressing glands exhibit high dp-ERK (A) and pAKT (B). Other oncogenes do not induce dp-ERK or pAKT unless there is a basal extrusion (arrow). (Note gain was increased to show even low, near-background levels of staining. Sample sizes: Ras^{V12} N = 6 DP-ERK, N=2 pAKT at 5d, Myc+ E2f/Dp+P35, at 15d N =6, Yki*+Dp+P35 at 20d N = 10, and Yki^{WT}+P35 N= 14 at 20d, GFP+P35 N = 8 at 15d. Note that P-AKT staining was performed at 10 days.

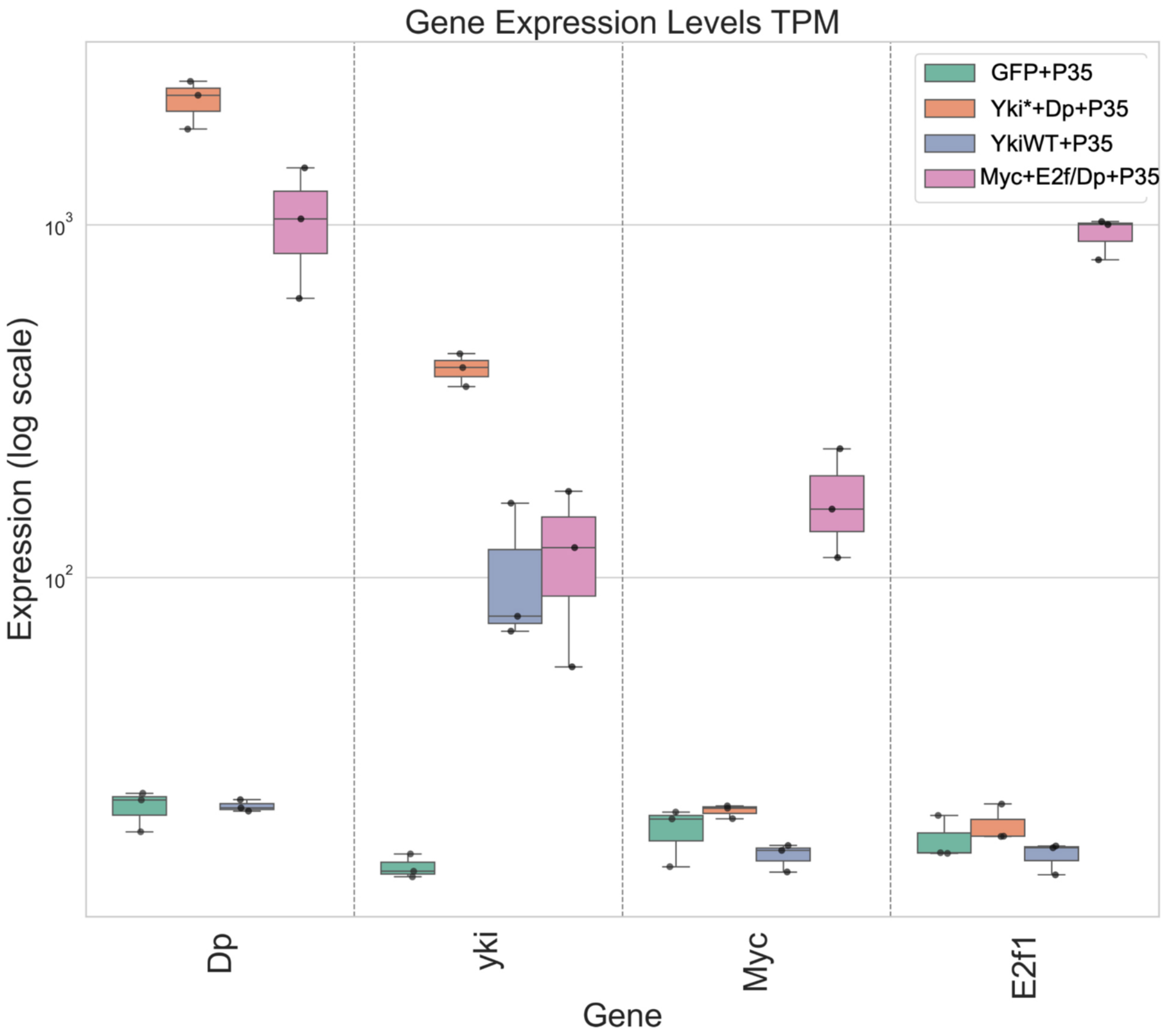

Fig. S6. Gene expression levels for the induced transgenes at 10d measured by RNAseq. Gene expression for the indicated genes is shown as transcripts per million (TPM) for each of 3 biological replicates.

Disease Models & Mechanisms • Supplementary information

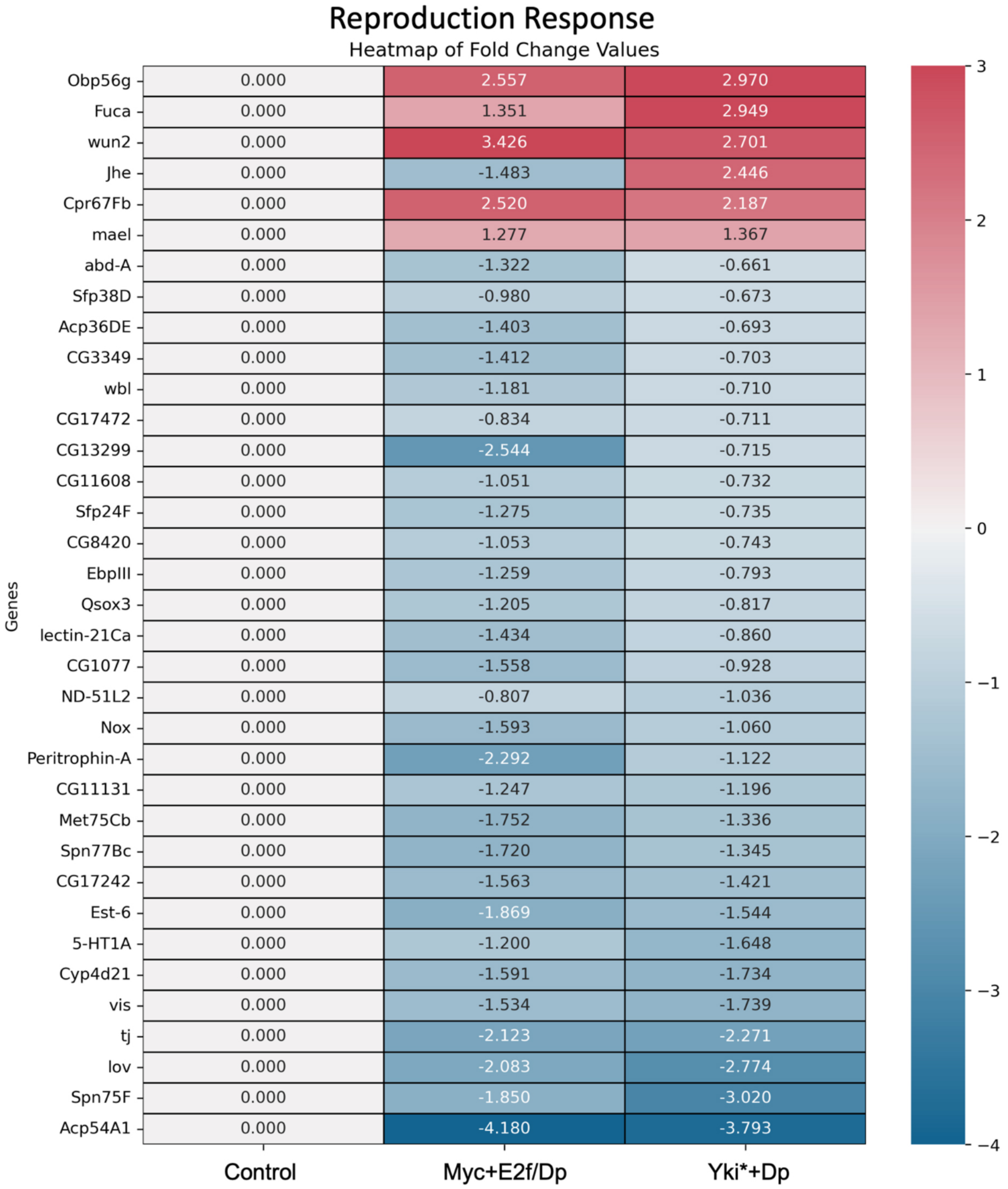

Fig. S7. Oncogenic activity reduces expression of genes associated with reproduction in the accessory gland. A heatmap of log2-based fold changes for genes involved in the biological process "reproduction response". These genes were selected based on a threshold of significance adj p-value < 0.05 cutoff and $\log_2$ fold change >1 or <-0.66 for both Yki*+Dp+P35 and Myc+E2F/Dp +P35 compared to controls expressing GFP+P35. The reproduction response gene list are from AmiGO.

Disease Models & Mechanisms • Supplementary information

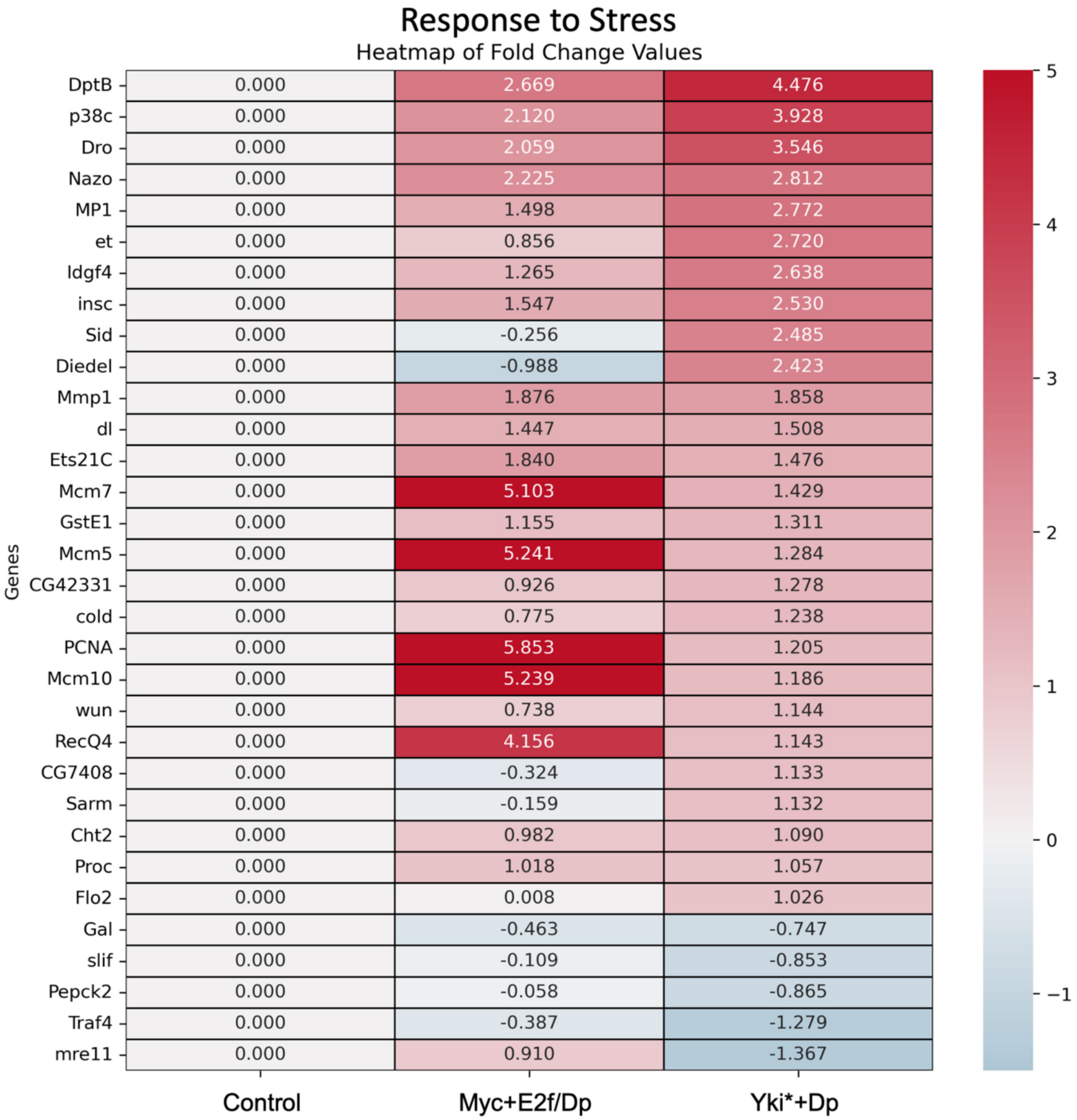

Genes	Control	Myc+E2f/Dp	Yki*+Dp
DptB	0.000	2.669	4.476
p38c	0.000	2.120	3.928
Dro	0.000	2.059	3.546
Nazo	0.000	2.225	2.812
MP1	0.000	1.498	2.772
et	0.000	0.856	2.720
Idgf4	0.000	1.265	2.638
insc	0.000	1.547	2.530
Sid	0.000	-0.256	2.485
Diedel	0.000	-0.988	2.423
Mmp1	0.000	1.876	1.858
dl	0.000	1.447	1.508
Ets21C	0.000	1.840	1.476
Mcm7	0.000	5.103	1.429
GstE1	0.000	1.155	1.311
Mcm5	0.000	5.241	1.284
CG42331	0.000	0.926	1.278
cold	0.000	0.775	1.238
PCNA	0.000	5.853	1.205
Mcm10	0.000	5.239	1.186
wun	0.000	0.738	1.144
RecQ4	0.000	4.156	1.143
CG7408	0.000	-0.324	1.133
Sarm	0.000	-0.159	1.132
Cht2	0.000	0.982	1.090
Proc	0.000	1.018	1.057
Flo2	0.000	0.008	1.026
Gal	0.000	-0.463	-0.747
slif	0.000	-0.109	-0.853
Pepck2	0.000	-0.058	-0.865
Traf4	0.000	-0.387	-1.279
mre11	0.000	0.910	-1.367

Fig. S8. Oncogenic activity increases expression of genes associated with a response to stress in the accessory gland

A heatmap of log_2-based fold changes for genes involved in the biological process “response to stress”. These genes were selected based on a threshold of significance adj p-value < 0.05 cutoff and log_2 fold change >1 or <-0.66 for both Yki*+Dp+P35 and Myc+E2F/Dp+P35 compared to controls expressing GFP+P35. The response to stress gene list are from AmiGO.

Disease Models & Mechanisms • Supplementary information

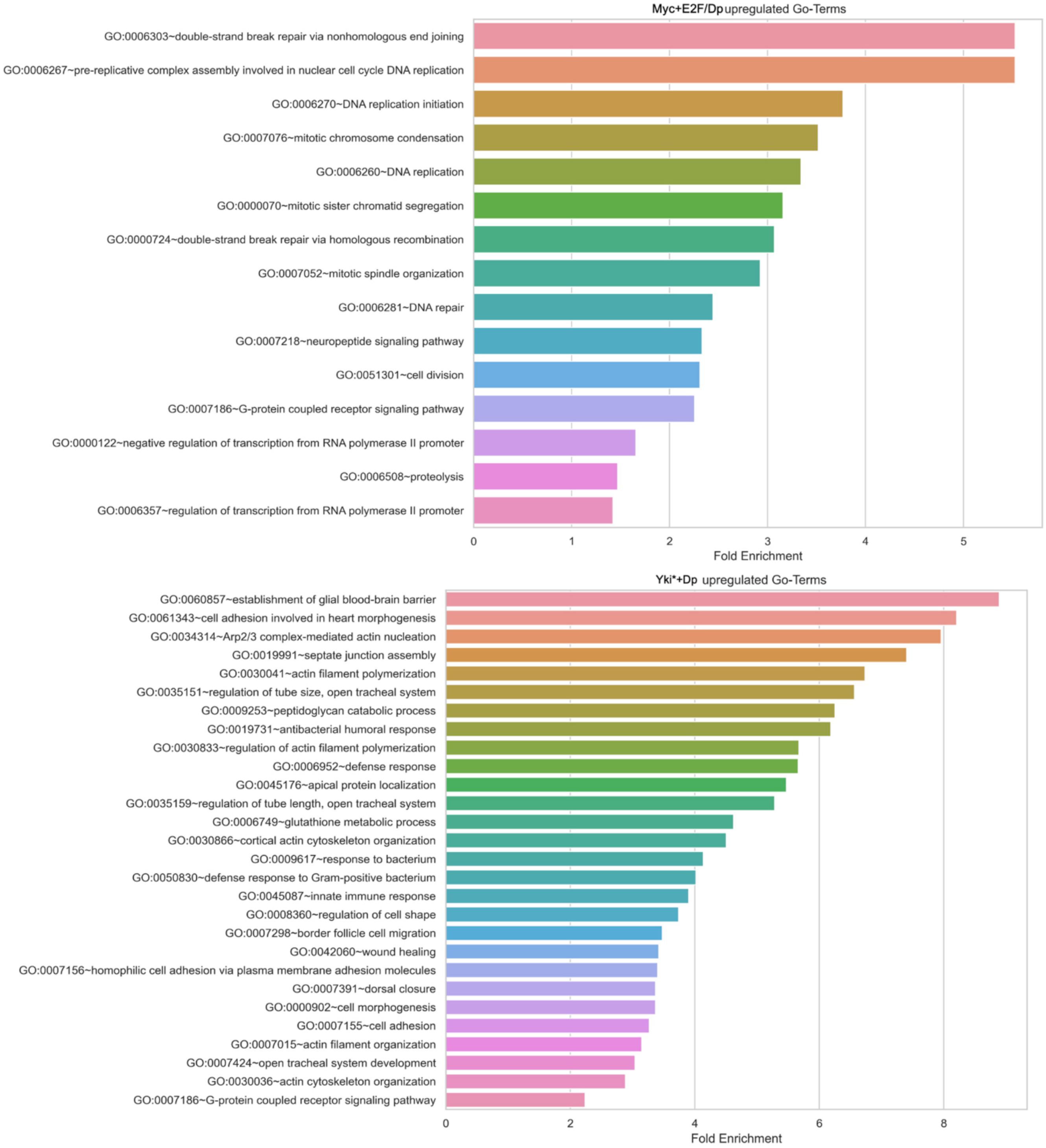

Fig. S9. GO term enrichment analysis

GO term enrichment analysis was performed for the upregulated genes in the indicated genotypes compared to controls (expressing GFP+P35 only) using DAVID and multiple redundant terms were collapsed using ReviGO. Analysis was limited to genes with an adj p-value of 0.05 and $\log_2$ fold changes of >1.

Disease Models & Mechanisms • Supplementary information

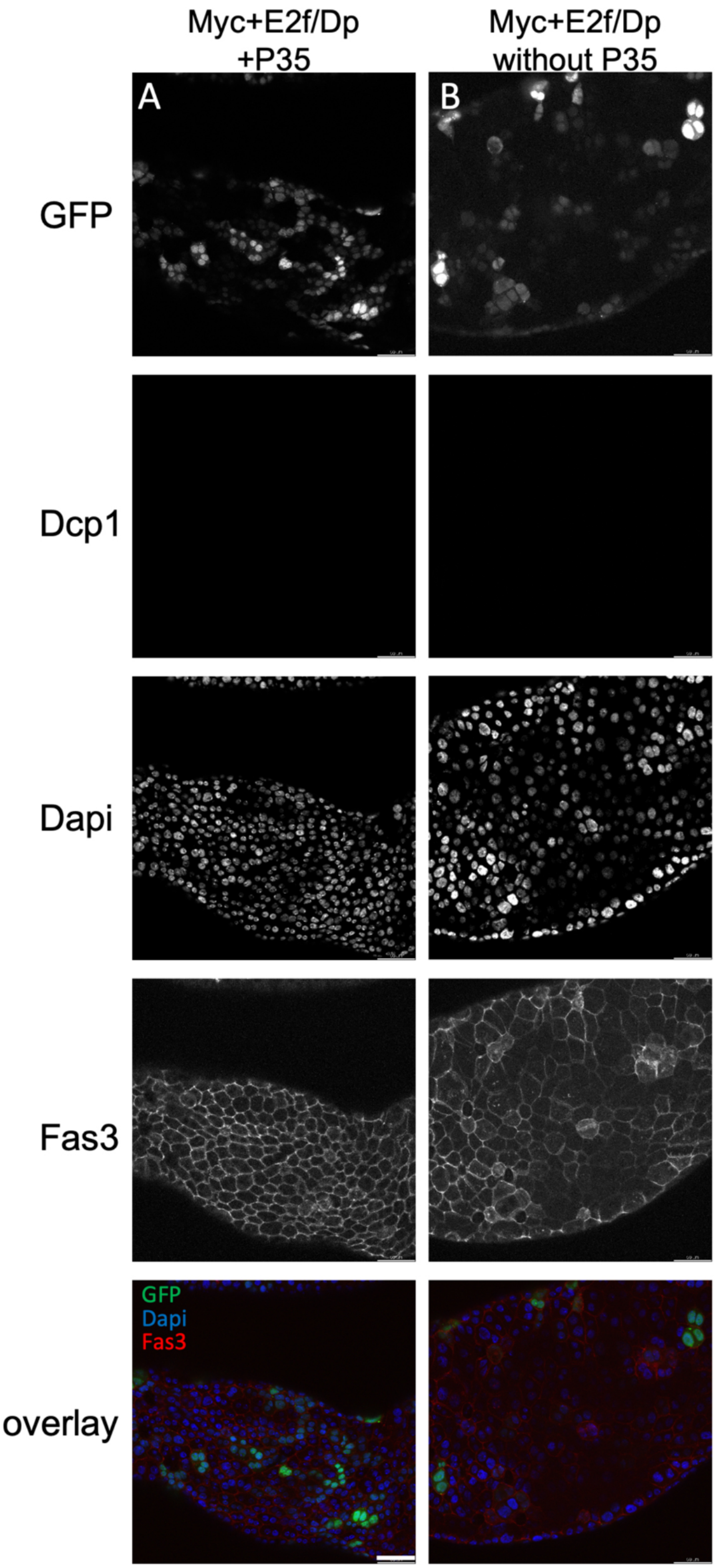

Fig. S10. Myc+E2f/Dp-induced hypertrophy does not require P35 and no cell death is observed in the absence of oncogenic activity without P35. Adult glands expressing the indicated transgenes at 10d were stained for DCP-1 and Fas3. No cell death is observed with or without P35 and hypertrophy and Fas3 mislocalization is observed in the absence of P35. N=6 for each genotype. Scale bar =50μm.

Disease Models & Mechanisms • Supplementary information

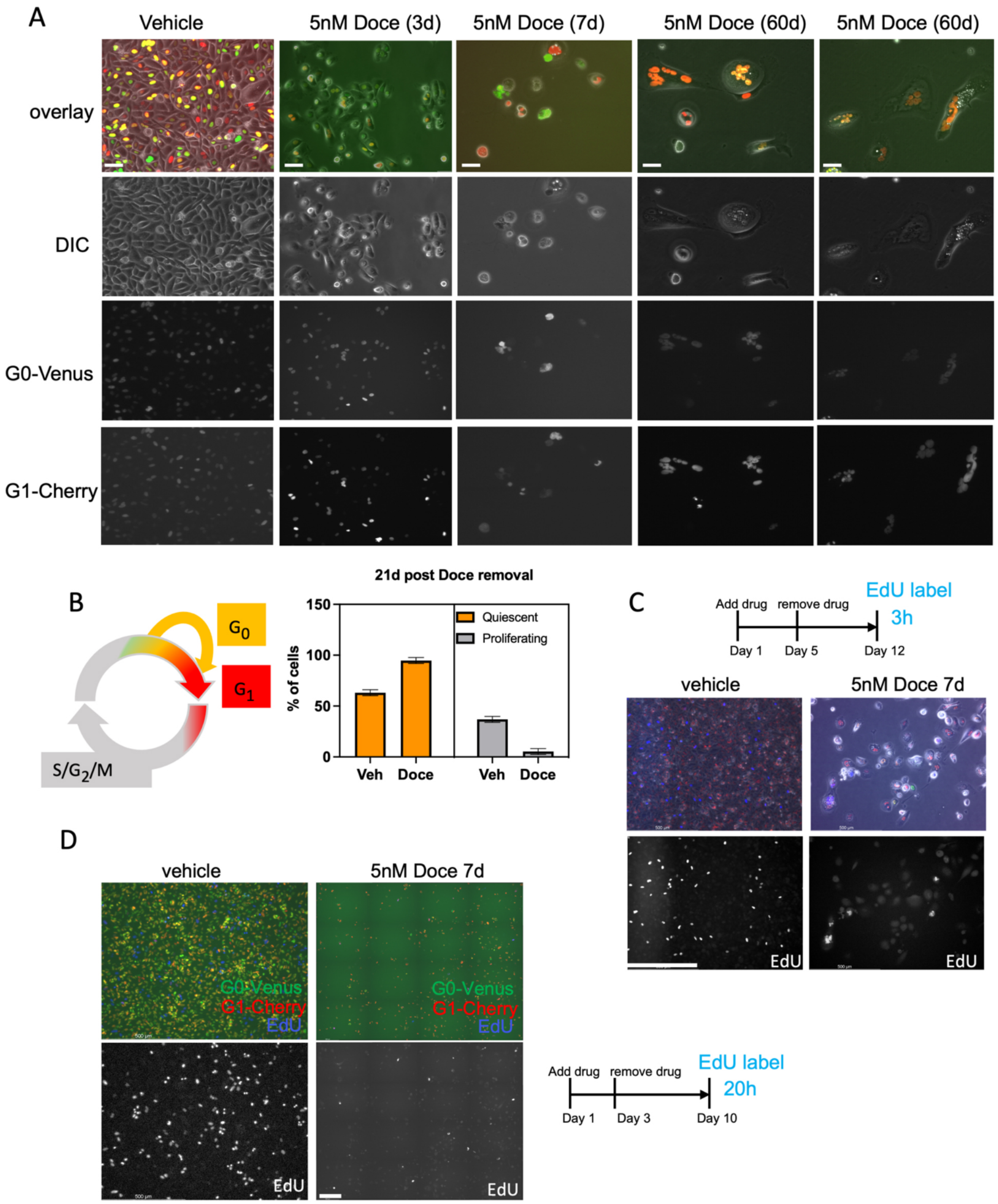

Fig. S11. PC3 cells that survive Docetaxel treatment form large, polyploid cells.
A. Cultured PC3 cells stably expressing cell cycle reporters p27K-Venus (G0) and Cdt1-Cherry (G0/G1), were treated with vehicle only (DMSO) or 5nM Docetaxel for 3 days, after which most PC3 cells die. After drug removal, long-term surviving PC3 cells were observed for the indicated number of days (3-60) and continue to grow. B. Using the p27K-Venus (G0) and Cdt1-Cherry (G0/G1) cell cycle reporters, we observe most long-term surviving poly-aneuploid PC3 cells become quiescent. Note the vehicle treated PC3 cells at 21d are confluent and therefore also exhibit high levels of quiescence. EdU labeling for 3h (C) or 20h (D) reveals low levels of endoreduplication. Note that the docetaxel treated sample in D is a tile scan to include more enlarged cells. The scale bars in D indicate the relative scales of the vehicle control panel vs the Docetaxel treated panel. Scale bars in A= 50μm. C, D = 500μm.

Disease Models & Mechanisms • Supplementary information

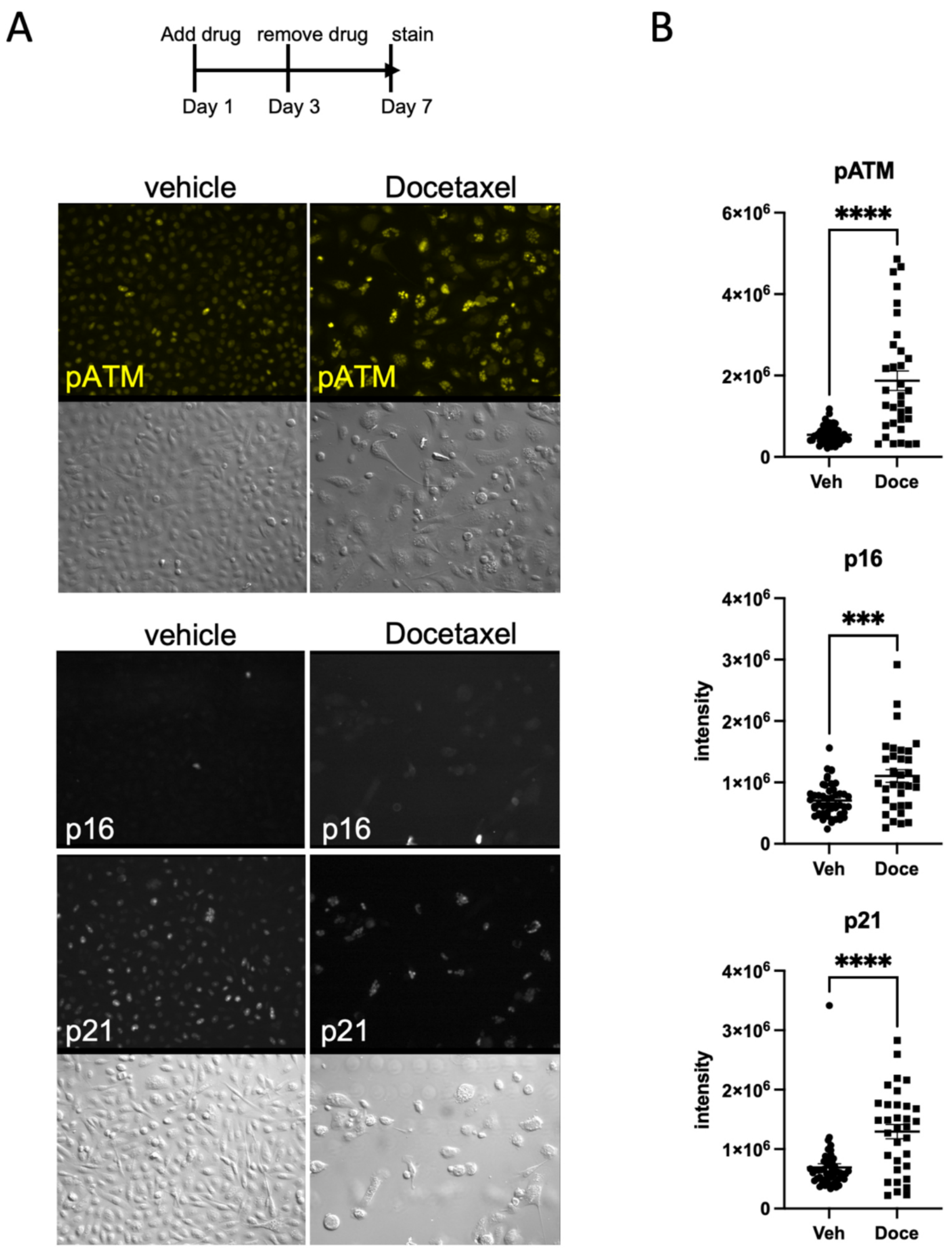

Fig. S12. Surviving poly-aneuploid PC3 cells express markers of cell cycle arrest and DNA damage. A. Cultured PC3 cells were treated with vehicle only (DMSO) or 5nM Docetaxel for 3 days and 5 days after drug removal (7 days total), stained for markers of cell cycle arrest and DNA damage. Poly-aneuploid cells exhibit increased pATM, p21 and p16, markers of DNA damage induced cell cycle arrest. B. Mean fluorescence intensity with background correction for >30 cells for each sample in panel A. Statistics are a t-test comparison with Welch's correction.

Disease Models & Mechanisms • Supplementary information

Table S1. Line plot data of Coracle fluorescence intensity in accessory gland epithelium (Fig. 3).

Available for download at
https://journals.biologists.com/dmm/article-lookup/doi/10.1242/dmm.052001#supplementary-data

Table S2. Quantification of nuclear intensity in GFP+ vs non-GFP+ cells from *Drosophila* accessory glands (Fig. 6).

Available for download at
https://journals.biologists.com/dmm/article-lookup/doi/10.1242/dmm.052001#supplementary-data

Table S3. Comparative gene expression profiles: *Drosophila* prostate-like endocycling cells vs polyaneuploid cancer cells.

Available for download at
https://journals.biologists.com/dmm/article-lookup/doi/10.1242/dmm.052001#supplementary-data

Table S4. qRT-PCR primer sequences used.

ITGB1	**Forward primer**	CCGCGCGGAAAAGATGAAT
	**Reverse primer**	CACATCGTGCAGAAGTAGGC
PTPN2	**Forward primer**	CAGTGTGAAGCTCTTGTCAGA
	**Reverse primer**	AGGGTTCAAGGAGCCAGATT
NOTCH2	**Forward primer**	CTCAACCTGCCTGGTTCCTA
	**Reverse primer**	GTGCTCCCTTCAAAACCTGG
CXCL8	**Forward primer**	AAAGACATACTCCAAACCTTTCCAC
	**Reverse primer**	CTCTGCACCCAGTTTTCCTTG
IL6	**Forward primer**	TACATCCTCGACGGCATCTC
	**Reverse primer**	CACCAGGCAAGTCTCCTCAT

Disease Models & Mechanisms • Supplementary information

Table S5. Transcriptomic overlap and functional enrichment between *Drosophila* tumor, wound repair, and combined stress responses.

Article title	Reference
Cell polarity opposes Jak/STAT-mediated Escargot activation that drives intratumor heterogeneity in a Drosophila tumor model	PMID: 36709425
Renal NF-κB activation impairs uric acid homeostasis to promote tumor-associated mortality independent of wasting	PMID: 36029766
Shared enhancer gene regulatory networks between wound and oncogenic programs	PMID: 37133250
Ets21C, Fos and Ftz-F1 drives JNK-mediated tumor malignancy	PMID: 26398940
An Ectopic Network of Transcription Factors Regulated by Hippo Signaling Drives Growth and Invasion of a Malignant Tumor Model	PMID: 27476594
Promoter Proximal Pausing Limits Tumorous Growth Induced by the Yki Transcription Factor in Drosophila	PMID: 32737120
The Drosophila Duox maturation factor is a key component of a positive feedback loop that sustains regeneration signaling	PMID: 28753614

Supplementary references

o PMID: 28753614

Khan, S. J., Abidi, S. N. F., Skinner, A., Tian, Y., & Smith-Bolton, R. K. (2017). The Drosophila Duox maturation factor is a key component of a positive feedback loop that sustains regeneration signaling. PLoS Genetics, 13(7), e1006937. https://doi.org/10.1371/journal.pgen.1006937

o PMID: 32737120

Nagarkar, S., Wasnik, R., Govada, P., Cohen, S., & Shashidhara, L. S. (2020). Promoter proximal pausing limits tumorous growth induced by the Yki transcription factor in Drosophila. Genetics, 216(1), 67–77. https://doi.org/10.1534/genetics.120.303419.

o PMID: 27476594

Atkins, M., Potier, D., Romanelli, L., Jacobs, J., Mach, J., Hamaratoglu, F., Aerts, S., & Halder, G. (2016). An ectopic network of transcription factors regulated by Hippo signaling drives growth and invasion of a malignant tumor model. Current Biology, 26(16), 2101–2113. https://doi.org/10.1016/j.cub.2016.06.035

o PMID: 26398940

Külshammer, E., Mundorf, J., Kilinc, M., Frommolt, P., Wagle, P., & Uhlirova, M. (2015). The interplay among Drosophila transcription factors Ets21c, Fos,z and Ftz-F1 drives JNK-mediated tumor malignancy. Disease Models & Mechanisms, 8(10), 1279–1293. https://doi.org/10.1242/dmm.020719

o PMID: 37133250

Floc'hlay, S., Balaji, R., Stanković, D., Christiaens, V. M., Bravo González-Blas, C., De Winter, S., Hulselmans, G. J., De Waegeneer, M., Quan, X., Koldere, D., Atkins, M., Halder, G., Uhlirova, M., Classen, A.-K., & Aerts, S. (2023). Shared enhancer gene regulatory networks between wound and oncogenic programs. eLife, 12, e81173. https://doi.org/10.7554/eLife.81173

o PMID: 36029766

Chen, Y., Xu, W., Chen, Y., Han, A., Song, J., Zhou, X., & Song, W. (2022). Renal NF-κB activation impairs uric acid homeostasis to promote tumor-associated mortality independent of wasting. Immunity, 55(9), 1594–1608.e6. https://doi.org/10.1016/j.immuni.2022.07.022

o PMID: 36709425

Chatterjee, D., Cong, F., Wang, X.-F., Costa, C. A. M., Huang, Y.-C., & Deng, W.-M. (2023). Cell polarity opposes Jak/STAT-mediated Escargot activation that drives intratumor heterogeneity in a Drosophila tumor model. Cell Reports, 42(2), 112061. https://doi.org/10.1016/j.celrep.2023.112061

Disease Models & Mechanisms • Supplementary information
